# Virtual Screening for Dual Inhibitors of HDAC6 and MPO: A Multistep Approach Integrating Chemical Space Analysis and Molecular Docking

**DOI:** 10.1021/acsomega.6c04249

**Published:** 2026-10-16

**Authors:** Bárbara M. de Viveiros, Camilo H. S. Lima, Nelilma C. Romeiro

**Affiliations:** † Laboratório Integrado de Computação Científica—LICC, Universidade Federal do Rio de Janeiro—Centro Multidisciplinar UFRJ Macaé, Av. Aluizio da Silva Gomes 50, Macaé, Rio de Janeiro 27930-560, Brazil; ‡ Laboratório de Modelagem Molecular (LabMMol), Departamento de Química Orgânica, Instituto de Química, 28125 Universidade Federal do Rio de Janeiro, Avenida Athos da Silveira Ramos 149, Bloco A, Rio de Janeiro, Rio de Janeiro 21941-853, Brazil

## Abstract

Cardiovascular diseases (CVDs) remain the leading cause of mortality worldwide, accounting for ∼17.9 million deaths annually. Despite therapeutic advances, treatments face limitations, such as adverse effects, drug resistance, and high costs. CVDs involve complex biological networks and compensatory pathways that limit the efficacy of single-targeted therapies. In this context, the multitarget-directed ligand (MTDL) strategy has emerged as a promising approach to enhance therapeutic efficacy by modulating proteins in different biochemical pathways involved in disease progression. Thus, this work reports a virtual screening study aimed at identifying novel dual inhibitors targeting histone deacetylase 6 (HDAC6) and myeloperoxidase (MPO), two enzymes implicated in the pathophysiology of CVDs and associated risk factors, for which no multitarget inhibition studies have been reported to date. Both targets converge in exacerbating CVDs by driving vascular inflammation and impairing cardiac autophagy. In this way, a ligand- and structure-based virtual screening approach was employed to identify potential multitarget hit compounds. The selection of reference compounds for virtual screening was based on four strategies combining the lowest IC_50_ values, clinical status, and shared physicochemical properties between the inhibitors of both enzymes, selected from a chemical space analysis. Molecular similarity was assessed via the Tanimoto index using reference ligands followed by molecular docking. Molecules **11** and **16** emerged as putative dual inhibitors, exhibiting structural similarity and crucial active-site interactions with HDAC6 (Zn^2+^) and MPO (heme, His95, Arg239). These findings support further investigation of these molecules as potential lead compounds for a dual-target cardiovascular therapy.

## Graphical abstract

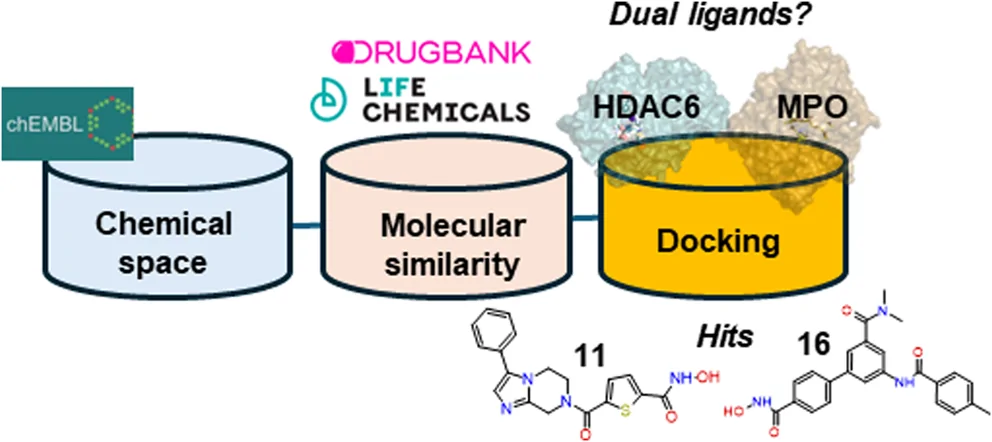

## 1 Introduction

Cardiovascular diseases (CVDs) remain a leading cause of global mortality, demanding therapies with improved efficacy, safety, and accessibility. Current pharmacological agents—including antihypertensives, statins, and antiplatelets—exhibit limitations such as adverse effects, drug resistance, and high costs, underscoring the need for innovative strategies.^1–3^

Recent research highlights the therapeutic potential of modulating dual targets within interconnected CVD pathways.^4,5^ Among these, histone deacetylase 6 (HDAC6) and myeloperoxidase (MPO) emerge as compelling candidates. Unlike most HDACs, HDAC6 resides essentially in the cytosol and is structurally distinguished by the presence of two homologous catalytic domains (CD1 and CD2).^6^ As a zinc-dependent metalloenzyme, its primary function is the deacetylation of α-tubulin.^7^ HDAC6 inhibitors exhibit a lower toxicity profile than *pan*-HDAC inhibitors, as their action is mostly restricted to the cytoplasm, preserving global gene transcription and reducing effects on nuclear targets.^8,9^ In addition to its established role in cell cycle regulation, recent evidence has expanded HDAC6’s field of action to cardiovascular physiology. Studies indicate that this enzyme plays a decisive role in modulating the elastic properties of the heart and diastolic function, making it a relevant target for the treatment of heart disease.^10,11^ Additionally, it was observed that HDAC6 inhibition increases autophagic flux in cardiomyocytes, a mechanism that correlates with tubulin hyperacetylation, suggesting that the induction of autophagy may underlie the observed cardioprotection.^12^ Given this scenario, HDAC6 is emerging as a versatile therapeutic target, with the potential to intervene not only in the regulation of cell proliferation but also in the management of cardiovascular pathologies. The central mechanism of this inhibition occurs at the enzyme’s catalytic site, where a zinc ion (Zn^2+^) is essential for deacetylation activity. To modulate this enzymatic pathway, drug design often relies on hydroxamic acids, molecules that act as Zinc-Binding Groups (ZBGs). Among the coordination modes described in the literature, the bidentate binding mode stands out as the canonical and most widely characterized mechanism in the rational design of these inhibitors.^13,14^ Some examples of selective HDAC6 inhibitors are described in Figure 1.^15^

**Figure 1:**
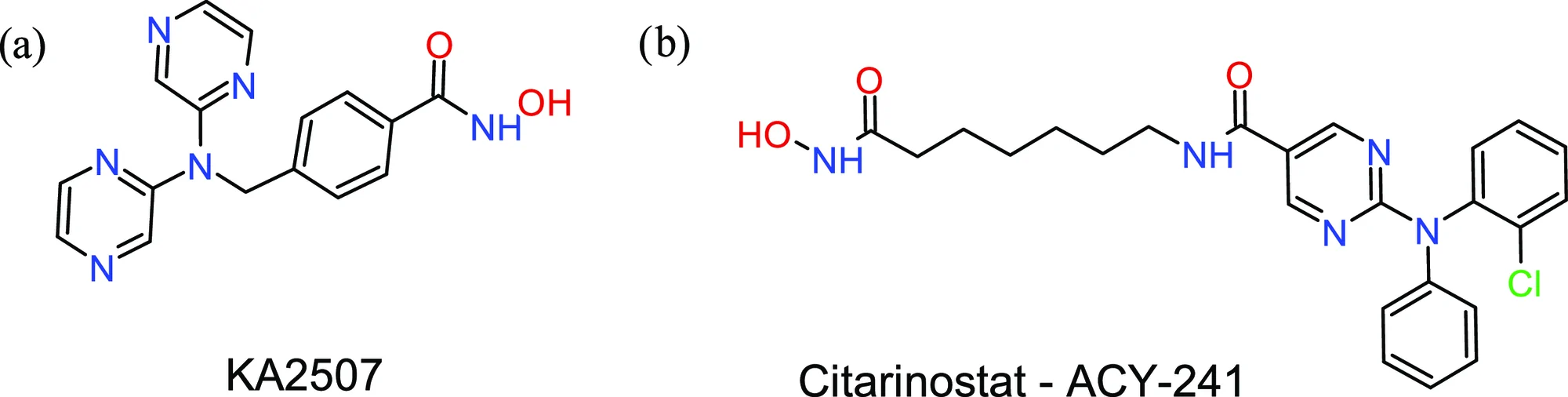
Examples of selective HDAC6 inhibitors: (a) KA2507; (b) citarinostat (ACY-241).

Myeloperoxidase (MPO), in turn, is a heme peroxidase expressed predominantly by neutrophils and plays a central role in the host’s innate immune defense by generating various reactive species essential for antimicrobial activity.^16^ The catalytic cycle of MPO begins with the native enzyme (in the ferric form) reacting with hydrogen peroxide to form Compound I, an iron-oxo intermediate with a high redox potential. From this state, MPO can follow two pathways: (a) the halogenation cycle, in which Compound I promotes a two-electron oxidation of chloride to form hypochlorous acid, regenerating the native enzyme; or (b) the peroxidation cycle, in which Compound I abstracts an electron or a hydrogen atom from a substrate. This process generates a free radical and forms Compound II (an iron­(IV) species). Finally, Compound II is reduced by a second substrate, completing the cycle by regenerating the native enzyme.^17–19^ However, this same oxidative capacity, when exacerbated, contributes significantly to tissue damage during inflammatory processes.^16^ In the context of CVDs, MPO has been widely studied due to the strong correlation between its elevated circulating levels and the increased risk of mortality and adverse outcomes.^16^ Evidence indicates that this enzyme is linked to conditions such as coronary artery disease and atherosclerosis.^20^ Consequently, MPO is recognized as a ″high-risk″ biomarker for patients diagnosed with acute coronary syndrome, heart failure, hypertension, and stroke, consolidating itself as a multifaceted therapeutic target in cardiovascular protection.^21^ Beyond its immune function, MPO oxidative byproducts interact with lipids, proteins, and nucleic acids, promoting tissue damage associated with chronic inflammatory states, such as atherosclerosis. The clinical relevance of this interaction is evidenced by the presence of MPO in human atheromatous plaques and by its colocalization with low-density lipoproteins (LDLs) oxidized by hypochlorous acid (HOCl) in atherosclerotic lesions.^22^ Some MPO inhibitors are shown in Figure 2. Irreversible inhibitors, upon being oxidized by MPO’s compound I, are converted into highly reactive free radicals. These radicals irreversibly modify the porphyrin ring of the heme group or bind covalently to the central iron atom, rendering the enzyme inert. In this context, 4-aminobenzoic acid hydrazide (4-ABAH) is among the most potent and widely used irreversible inhibitors in laboratory studies.^18,23^ On the other hand, thiophene and thiourea derivatives (*e.g*., PF-06282999) are inhibitors that inactivate MPO by single-electron abstraction, followed by covalent modification of the heme cofactor.^17^ Also, reversible MPO inhibitors have been reported, which, unlike the former, do not cause permanent damage to the enzyme’s structure. Instead, these compounds act by physically competing with MPO substrates, such as chloride and hydrogen peroxide, to occupy the active site and block access to the heme group. For example, salicylhydroxamic acid (SHA) was one of the first reversible MPO inhibitors identified, although characterized by weak potency.^19,24^ Other notable examples are triazolopyridines, such as 7-benzyl triazolopyridine,^17^ which offer high affinity and acid stability, avoiding direct coordination with iron to minimize both toxicity and the risk of heme-mediated oxidation.

**Figure 2:**
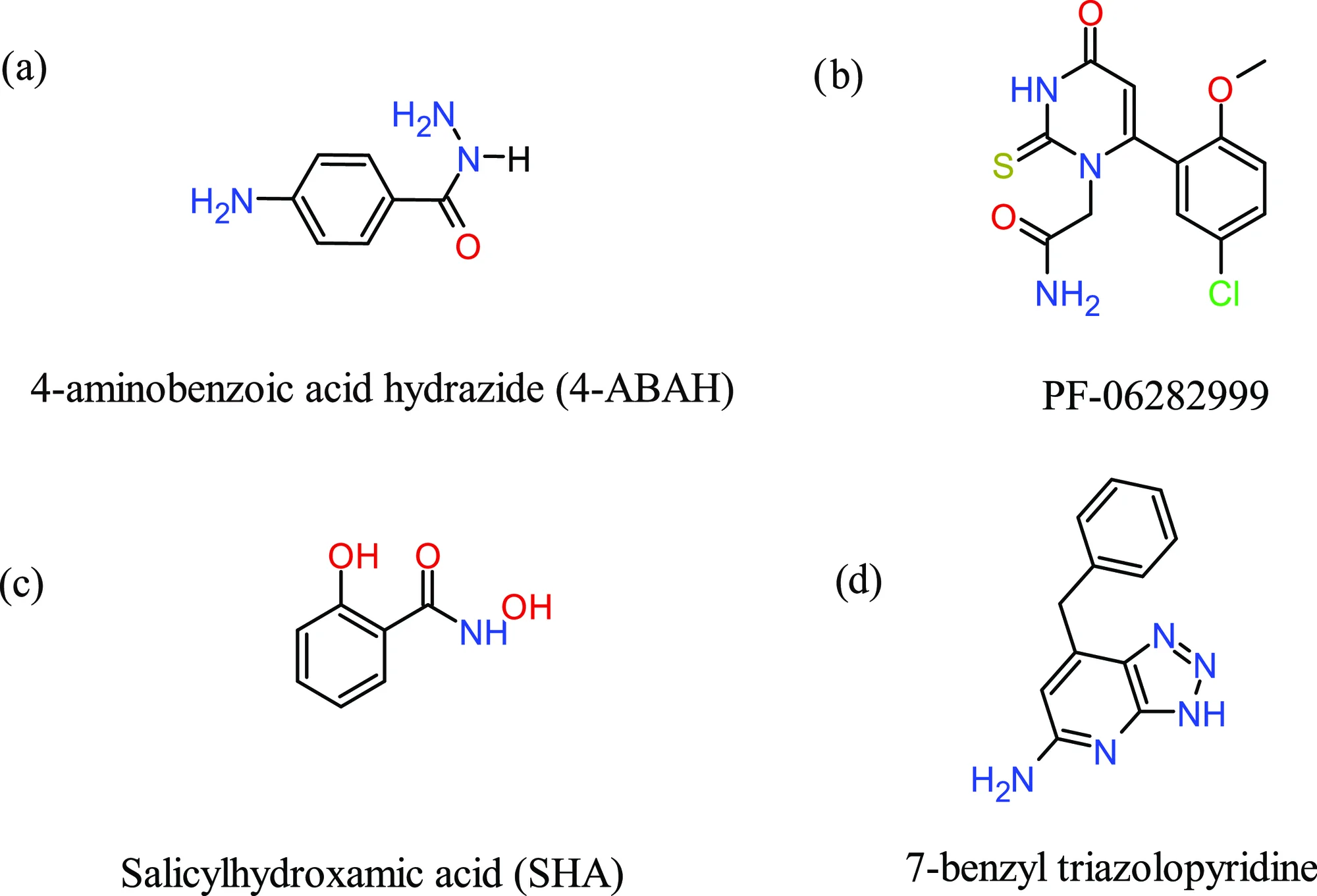
Examples of MPO inhibitors: (a) 4-aminobenzoic acid hydrazide (4-ABAH); (b) PF-06282999; (c) salicylhydroxamic acid (SHA); and (d) 7-benzyl triazolopyridine.

Although HDAC6 and MPO possess distinct structures, biological functions, and catalytic mechanisms and despite the challenges associated with simultaneously targeting both enzymes, their dual inhibition may represent a potential therapeutic strategy for the treatment of CVDs. Critically, both targets are implicated in overlapping inflammatory and fibrotic cascades, suggesting that concurrent inhibition could yield synergistic benefits that surpass those of single-target approaches.

Multitarget drug discovery, however, faces significant challenges in balancing potency and selectivity across distinct proteins.^25,26^ To address this, an integrated *in silico* strategy was employed to preliminarily evaluate potential dual HDAC6/MPO inhibitor candidates, a simultaneous targeting strategy for these two enzymes that has not been reported previously. The workflow began by prioritizing highly active reference compounds through chemical space analysis by using physicochemical descriptors. This approach leverages cheminformatics to efficiently navigate chemical space, prioritizing scaffolds with predicted activity against both targets.^27,28^ Subsequently, ligand-based similarity virtual screening and structure-based molecular docking were conducted to study theoretical interactions, demonstrating the feasibility of the proposed dual-target approach and providing a structural starting point for further development.

## 2 Materials and Methods

### 2.1 Experimental Section

#### 2.1.1 Selection Strategy for the Obtention of Reference HDAC6 and MPO Inhibitors

Previously reported inhibitors of the targets HDAC6 (ChEMBL ID: CHEMBL1865) and MPO (ChEMBL ID: CHEMBL2439) were retrieved from the ChEMBL database (release 35; accessed Jan 06, 2025).^29^ The inclusion criteria were defined as follows: human organism (Target organism: *Homo sapiens*), single protein targets (Target Type: single protein), and compounds with defined IC_50_ values ≤ 10 μM. Entries with undefined activity values and duplicate compounds were excluded from the data set.

From this filtered set, compounds for virtual screening were selected and refined by using four progressive methodological strategies. Initially (Strategy I), 30 molecules were selected based solely on the lowest absolute IC_50_ values for each target, HDAC6 (Table S1) and MPO (Table S2). Next (strategy II), a subgroup from this first stage was filtered and grouped by high similarity in the reduced physicochemical space using a principal component analysis (PCA) model focused on the properties of the inhibitors for both studied targets (Section 2.1.2). Subsequently, (strategy III), a hybrid set was developed, by integrating the 15 molecules with the lowest IC_50_ values with 15 inhibitors in advanced clinical stages, and the clinical validation was confirmed by consulting the DrugBank database. Therefore, a total of 30 compounds were prioritized as HDAC6 (Table S3) and MPO (Table S4) reference compounds. Finally, in strategy IV, this hybrid set underwent a final refinement by reapplying the physicochemical selection filter established by the PCA model (Section 2.1.2).

#### 2.1.2 Selection of Reference Compounds from the Comparative Study of the Chemical Space of HDAC6 and MPO Inhibitors via PCA for Strategies II and IV

In this study, the workflow depicted in Figure 3, developed by the authors using KNIME 5.9.0,^30^ was employed to analyze the chemical space occupation of known inhibitors targeting the proteins under investigation. The following molecular descriptors were used: molecular weight (MW), number of hydrogen-bond donors (nHBDon) and acceptors (nHBAcc), topological polar surface area (TopoPSA), number of rotatable bonds (nRotB), and a computational estimate of the logarithm of the octanol–water partition coefficient (ALogP). The descriptors were standardized using the Z-score (Gaussian) statistical transformation within the KNIME workflow.

**Figure 3:**
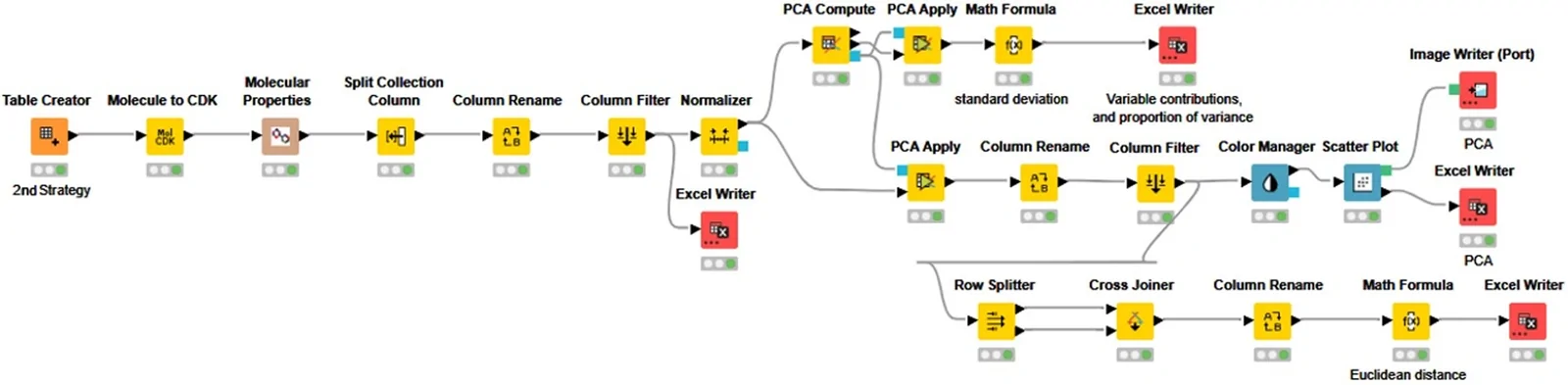
KNIME workflow used for the study of the chemical space based on molecular properties. A description of each KNIME node used in the chemical space workflow can be found in the Supporting Information, Table S5.

Using the workflow developed in KNIME, principal component (PC) values were generated from previously calculated molecular properties. Dimensionality reduction was performed using Principal Component Analysis (PCA), in which the feature set was projected onto a two-dimensional space defined by the first two principal components (PC1 and PC2). Then, an analysis of the contribution of each molecular property to the formation of each principal component and its respective variance was performed. Finally, the Euclidean distance for each point in the KNIME-generated plot (eq 1) was calculated, and a sensitivity analysis of the data was performed.

1 $$d\left(x,y\right)=\sqrt{\sum_{k=1}^{n}{\left(x_{k}-y_{k}\right)}^{2}}$$

Eq 1: Euclidean distance calculation formula.

The final selection of compounds was based on those with distances between points lower than 0.5, aiming to identify the most similar compounds for both targets while preserving approximately 50% of the reference compounds from the overall strategy (strategies I and III).

#### 2.1.3 Similarity-Based Compound Selection

Based on the selection of known inhibitors of the target proteins in the previous step, a molecular similarity study was conducted using compounds retrieved from the LIFECHEMICALS (https://lifechemicals.com/)^31^ and Drugbank (https://go.drugbank.com/)^32^ databases, employing the KNIME bioinformatics platform (https://www.knime.com/) as shown in Figure 4. The workflow developed for the molecular similarity study was structured according to previously published workflows on the KNIME Hub.^33,34^ Molecular similarity was assessed by using the Tanimoto index. Similarity calculations were performed using MACCS-type fingerprints, and a cutoff of ≥75% was used to select compounds. The selection of substances for the subsequent stages was carried out using a few exclusion criteria, including previously reported inhibition in the ChEMBL^29^ and PubChem^35^ databases and duplicate values.

**Figure 4:**
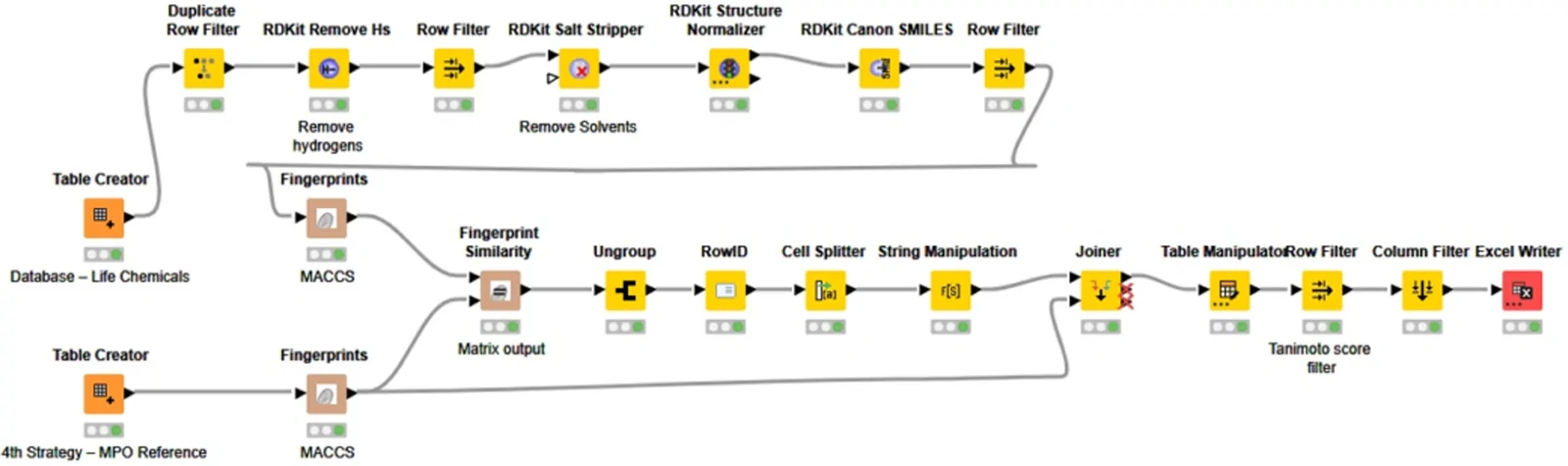
KNIME workflow used for the similarity study. A description of each KNIME node used in the similarity study workflow can be found in the Supporting Information, Table S6.

#### 2.1.4 Docking Studies

The three-dimensional structures of the proteins used for the docking study were obtained from the Protein Data Bank (PDB)^36^ and processed in PyMOL (version 0.99).^37^ The structures were loaded into the program, and ligands or cofactors were removed. Subsequently, the chain of interest was selected, retaining only the portion corresponding to the analyzed protein. The 3D structures of HDAC6 (PDB ID: 5EDU^38^) and MPO (PDB ID: 6WY7 ^17^), with resolutions of 2.79 and 2.09 Å, respectively, were used in the process. For PDB ID: 5EDU, the protonation states of ionizable residues were determined at pH 7.4 using the H++ server.^39^ For the Zn^2+^ ion, a formal charge of +2 was explicitly assigned. For the MPO structure, hydrogen atoms were added during the protein preparation stage using CCDC Hermes 2024.1.^40^ The docking studies were performed using GOLD 2024.1.0.^41^ For validation, redocking was performed using the GoldScore, ChemPLP, and ChemScore scoring functions, with binding site radii of 10, 15, and 20 Å for HDAC6 and MPO. For HDAC6, the nitrogen atom (ID 7663) of HIS610 was used as the reference point for defining the binding site space, whereas for MPO, the iron atom (ID 4576) of the heme group served as the spatial reference. The ligands were converted to 3D in MolView (https://molview.org/)^42^ and hydrogen additions were performed at pH 7.4 using Avogadro 1.1.1 (https://avogadro.cc/).^43^ The docking study was performed in triplicate, and the obtained score values were used to calculate the standard deviation. The best resulting poses and surface contours were analyzed with Discovery Studio 2016.^44^ Chemical structures were drawn using ChemSketch (Freeware) software from ACD/Laboratories 2024.2.3 (Advanced Chemistry Development, Inc.).^45^ Statistical analyses comparing the molecular docking scores for the reference and candidate compounds for MPO and HDAC6 were conducted in Microsoft Excel 365, using the *Real Statistics* add-in. Data normality was verified using the Shapiro-Wilk test, while homogeneity of variances was assessed using the F-test. For comparisons between groups with a normal distribution, Student’s *t*-test was employed in cases of homoscedasticity (equal variances), and Welch’s *t*-test was used in cases of heteroscedasticity (unequal variances). For non-normally distributed data, the nonparametric Mann–Whitney *U* test was applied. The significance level was set at 5% (*p* < 0.05).

## 3 Results and Discussion

The discovery of dual protein inhibitors has driven significant efforts to explore the chemical space. Advanced computational tools and methodologies, such as multitarget de novo design and docking strategies, have been developed to optimize the identification of these compounds.^46–48^ Initially, the search for previously reported inhibitors of targets HDAC6 and MPO was conducted using the ChEMBL database as described in Section 2.1.1. The search retrieved 19,410 HDAC6 inhibitors and 941 MPO inhibitors, respectively. Subsequently, a filtering step was applied to select inhibitors with IC_50_ values ≤ 10 μM, yielding 4518 for HDAC6 and 441 for MPO.

From this search, the selection of reference compounds was conducted through four methodological strategies (described in Sections 2.1.1 and 2.1.2). The intention was to compose a set with broad structural diversity that captures the inherent characteristics of HDAC6 and MPO inhibitors, with the aim of facilitating the future identification of dual inhibitor candidates. Furthermore, the second and fourth strategies employed chemical space analysis to map the physicochemical intersections between the inhibitors of both targets. This approach allowed refining the group previously selected in strategies 1 and 3 (based on low IC_50_ values), prioritizing molecules that, although targeting structurally different receptors, shared highly similar properties. Thus, it was possible to obtain references with strict, compatible limits on size, lipophilicity, and flexibility, thereby defining a similar profile for both of the biological scenarios. The ultimate goal was to employ these compounds in the subsequent virtual screening step, guiding the search and selection of new analogues within this same mapped chemical space.

An important limitation of the search for the lowest IC_50_ values in the ChEMBL database for selecting reference compounds is the heterogeneity of these data, which derive from a broad scientific literature employing different biochemical assay formats. Consequently, the direct comparison of these IC_50_ values on a standardized numerical scale may present limitations in “lowest IC_50_” rankings. A possible mitigation for the impact of this experimental variability is that Strategies 3 and 4 did not rely solely on the lowest IC_50_ values as a cutoff criterion but also included the selection of reference compounds with advanced clinical studies, even with higher IC_50_ values. Thus, although the ranking based on ChEMBL data should be used with caution regarding its original experimental sources to ensure greater methodological rigor, it was expected that combining it with the compounds’ clinical status would confer greater robustness to the final selection of these compounds.

In the first reference selection strategy, the 30 compounds with the lowest IC_50_ values were selected for each target individually, HDAC6 (Table S1) and MPO (Table S2). The second strategy consisted of selecting the compounds with the highest structural similarity between the inhibitors of targets HDAC6 and MPO, based on the analysis of the chemical space shared by these compounds. In the third selection strategy, considering the presence of different classes of compounds in the ChEMBL database—including experimental molecules and known drugs—a total of 30 compounds were prioritized for HDAC6 (Table S3) and MPO (Table S4). Of these, 15 correspond to compounds with the lowest IC_50_ values, regardless of classification, whereas the remaining 15 refer specifically to approved drugs or molecules with more advanced studies that also exhibit low IC_50_ values. Finally, the fourth strategy employed chemical analysis of the molecules selected in the third strategy to identify the most structurally similar compounds across the different receptors, aiming to prioritize potential dual-inhibition candidates.

A workflow was developed for the second and fourth strategies, and the chemical space was explored using KNIME. Some of the properties used in the chemical space study (nHBDon, nHBAcc, TopoPSA, nRotB, and ALogP) were selected based on the parameters defined by Lipinski’s Rule of Five,^49^ which establishes empirical criteria for predicting the oral absorption of small molecules. This approach has previously been reported in chemical space studies for inhibitor characterization.^50^

Next, the principal components (PCs) were calculated along with the contribution of each chemical property to the formation of each PC and its variance. This resulted in a PCA plot, with the representation of the chemical space of all compounds selected in the second strategy (Figure 4). Furthermore, the contributions of molecular descriptors to the principal components were assessed, enabling the identification of the most relevant properties for distinguishing the chemical profiles of the inhibitors for each target. The developed workflow constitutes a robust platform for chemical space exploration and supports the rational prioritization of compounds in early stages of drug discovery. From the analysis performed using the developed workflow, a PCA plot (Figure 5) was obtained, representing the chemical space of all compounds selected in the first strategy.

**Figure 5:**
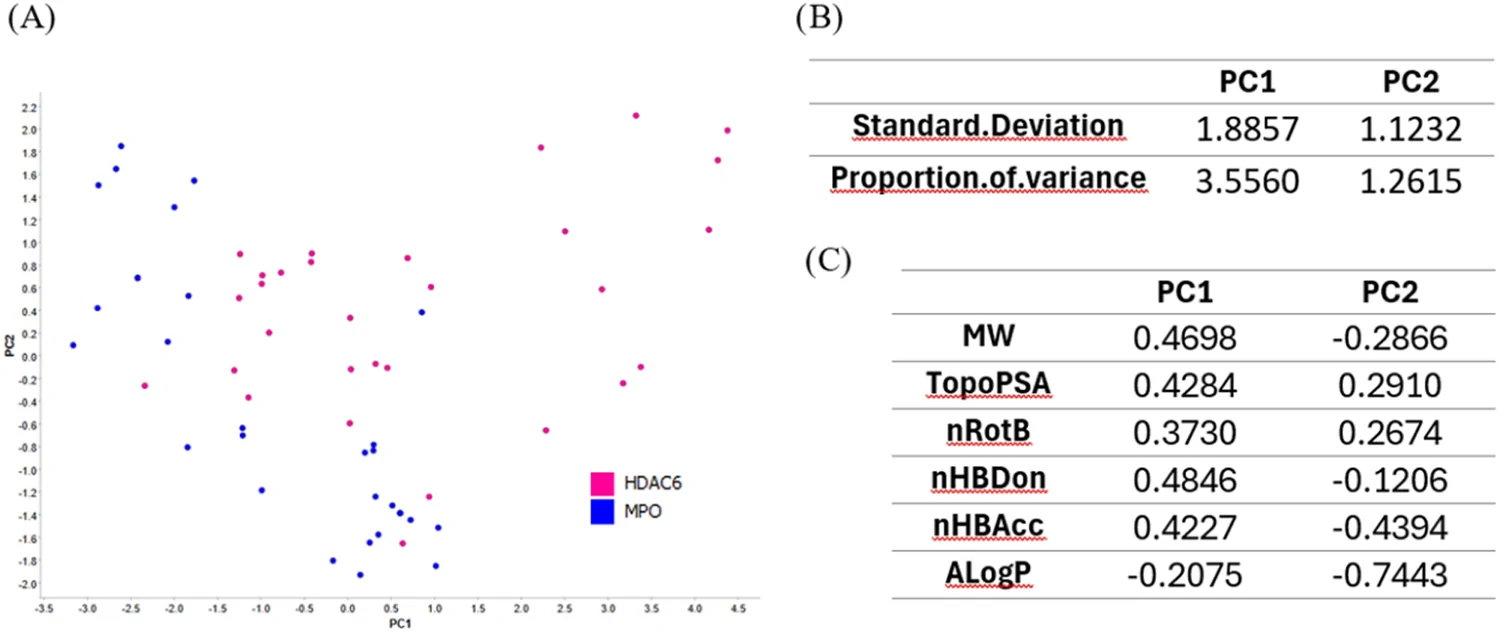
(A) PC1 vs PC2 plot with the representation of the chemical space of the reference HDAC6 and MPO inhibitors, according to the second strategy. (B) Standard deviation and proportion of variance explained by PC1 and PC2. (C) Variable contributions to the formation of PC1 and PC2.

From the plot in Figure 5, it is evident that the PC1 vs. PC2 analysis accounted for approximately 80.3% of the variance in the sampled chemical space. Additionally, MW, nHBDon, TopoPSA, and nHBAcc contribute most significantly to PC1, whereas AlogP contributes predominantly to PC2.

Subsequently, an analysis of the most similar compounds among MPO and HDAC6 inhibitors was conducted by using the PC1 and PC2 values obtained. Using Euclidean distance calculations, a sensitivity analysis was conducted to evaluate different cutoff thresholds for compound selection (Table S7). The objective was to balance selecting similar reference compounds with limiting excessive sample expansion given that data from a broader sampling had already been addressed in the first strategy. Therefore, the 0.5 threshold was selected based on pairwise similarity mapping and preservation of structural diversity. The application of this threshold in the second strategy resulted in a molecule recovery rate of 20% for HDAC6 and 50% for MPO, relative to the original set. Table 1 presents the molecules selected from this analysis, which constitute the reference compounds from the second strategy.

**Table 1:** Reference Molecules Selected from the Second Strategy

**number**	**HDAC6**	**MPO**
**1**	CHEMBL3670669	CHEMBL4751166
**2**	CHEMBL4177129	CHEMBL4068067
**3**	CHEMBL2018446	CHEMBL4878307
**4**	CHEMBL3670668	CHEMBL4877383
**5**	CHEMBL4531853	CHEMBL4482878
**6**	CHEMBL4536605	CHEMBL4752248
**7**		CHEMBL4861370
**8**		CHEMBL4795827
**9**		CHEMBL4763667
**10**		CHEMBL4453728
**11**		CHEMBL4594116
**12**		CHEMBL4531688
**13**		CHEMBL4747269
**14**		CHEMBL4851535
**15**		CHEMBL2346670

In the third strategy, the selection aimed to balance biological potency (low IC_50_ values) and chemical diversity to ensure structural representativeness without compromising the inhibitory potential. For this purpose, 15 compounds with the lowest IC_50_ values were selected, along with 15 molecules at more advanced clinical stages, with IC_50_ values within the established limits for the receptors HDAC6 and MPO (Tables S3 and S4). This approach focused on expanding the possibilities for identifying relevant compounds with structural representativeness for targets HDAC6 and MPO rather than concentrating on evaluating the selection methods themselves. For the fourth selection strategy, the chemical space representation of the 30 reference inhibitors for each receptor, obtained in the previous step, was studied. The PCA plot is shown in Figure 6.

**Figure 6:**
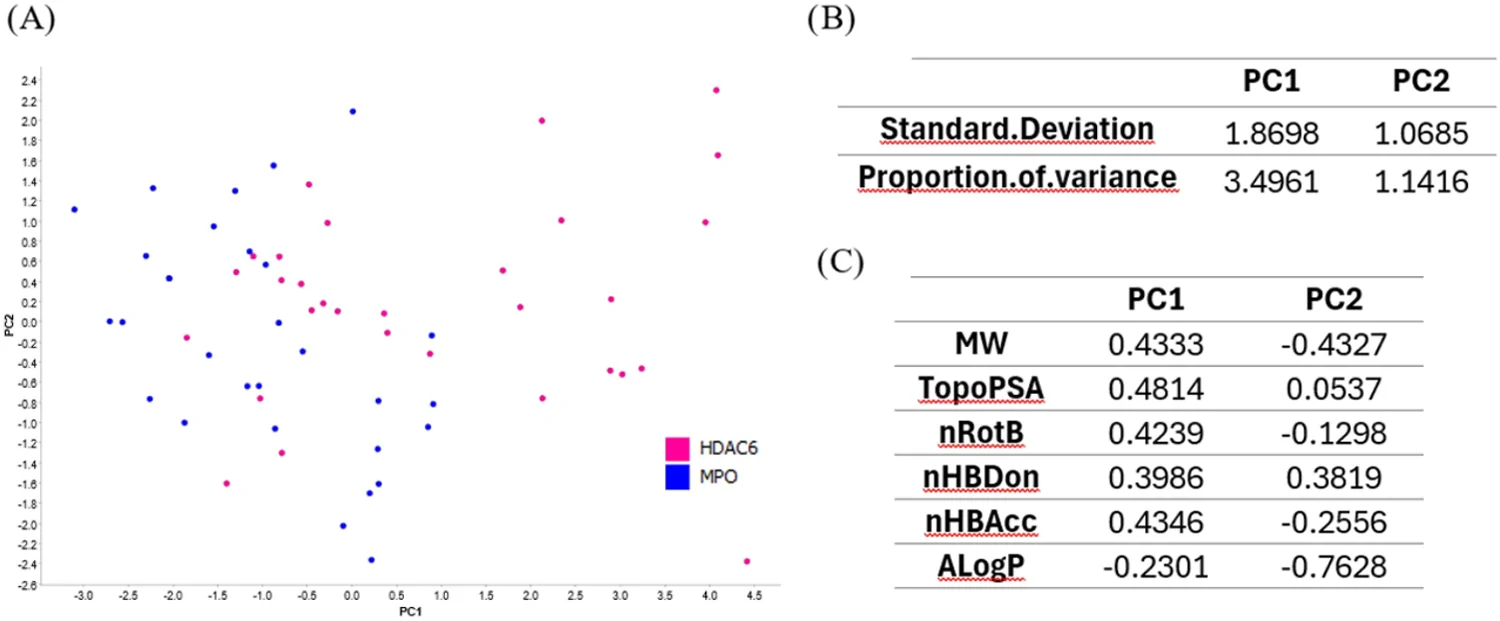
(A) PC1 vs PC2 plot with the representation of the chemical space of the reference HDAC6 and MPO inhibitors, according to the fourth strategy. (B) Standard deviation and proportion of variance explained by PC1 and PC2. (C) Variable contributions to the formation of PC1 and PC2.

From the analysis in Figure 6, it is evident that PC1 vs. PC2 accounted for 77.2% of the variance. In addition, TopoPSA, nHBAcc, MW, and nRotB contribute most significantly to PC1, while ALogP makes a greater contribution to PC2. The sensitivity analysis based on Euclidean distance calculations for the fourth strategy is presented in Table S8. Following the same rationale established in the second strategy, the application of the 0.5 threshold yielded recovery rates of 36.67% for MPO and 40.00% for HDAC6. From this fourth approach, 12 HDAC6 reference inhibitors and 11 MPO reference inhibitors were selected Table 2.

**Table 2:** Reference Molecules Selected from the Fourth Strategy

**number**	**HDAC6**	**MPO**
**1**	CHEMBL4104117	CHEMBL1043
**2**	CHEMBL99	CHEMBL4453728
**3**	CHEMBL4748025	CHEMBL2105120
**4**	CHEMBL27759	CHEMBL4068067
**5**	CHEMBL4863515	CHEMBL56367
**6**	CHEMBL487253	CHEMBL4548537
**7**	CHEMBL1789941	CHEMBL1945275
**8**	CHEMBL2018302	CHEMBL3633460
**9**	CHEMBL483254	CHEMBL86
**10**	CHEMBL98	CHEMBL150
**11**	CHEMBL96051	CHEMBL4861370
**12**	CHEMBL3912061	

In the next step, similarity analysis was performed between the molecular databases and the reference groups selected for each of the established strategies. From the most similar molecules, a quantitative evaluation was conducted for molecules with similarity greater than 75% in each database (DrugBank and Life Chemicals) for each receptor, along with the maximum similarity value obtained. These results are presented in Table 3.

**Table 3:** Similarity Study between the Reference Compounds and the Molecular Databases^a^

	**molecular database**	**receptor**	**maximum similarity** (%)	**number of molecules with ≥75% similarity**
**1** ^**st**^ **strategy**	DrugBank	HDAC6	84	29
	Life Chemicals		86	242
	DrugBank	MPO	89	172
	Life Chemicals		98	1382
**2** ^**nd**^ **strategy**	DrugBank	HDAC6	84	14
	Life Chemicals		86	184
	DrugBank	MPO	89	102
	Life Chemicals		89	677
**3** ^**rd**^ **strategy**	DrugBank	HDAC6	83	29
	Life Chemicals		84	150
	DrugBank	MPO	97	210
	Life Chemicals		98	1137
**4** ^**th**^ **strategy**	DrugBank	HDAC6	82	10
	Life Chemicals		81	29
	DrugBank	MPO	97	59
	Life Chemicals		98	346

a Molecular similarity was assessed using the Tanimoto index.

For the molecular docking study, the top five most similar molecules for each receptor were selected on the basis of each applied strategy, resulting in a total of 40 molecules (20 for MPO and 20 for HDAC6). Next, duplicate molecules across different strategies were removed, resulting in a final set of 27 unique molecules: 12 candidate ligands for HDAC6 and 15 for MPO. Subsequently, a new search was conducted using the available SMILES in the ChEMBL and PubChem databases for each selected molecule to identify and retain only those without prior studies related to HDAC6 or MPO. As a result, 17 molecules (numbered 1–17) without prior reports of investigation with the receptors of interest were retained and proceeded to the next step (see Supporting Information, Table S9). Although the virtual screening step employed structural similarity metrics to select potential hits, it is necessary to consider the possible presence of the activity cliffs. As established in the literature,^51^ minor modifications to the chemical scaffold can induce steric clashes or the loss of key contacts within the active site, thereby altering the target-ligand recognition profile. Therefore, ligand-based similarity alone does not guarantee a biological efficacy. This inherent limitation underscores the importance of integrating structure-based approaches to ligand similarity methods, and consequently, molecular docking was employed as an essential step to evaluate three-dimensional interactions and refine the selection of potential candidate compounds.

For molecular docking, the methodology was initially validated by redocking. The results are shown in Figure 7, for HDAC6 and MPO, respectively. For HDAC6, the CHEMPLP scoring function with a radius of 15 Å reported the best RMSD of 0.77, while for MPO, the best RMSD was obtained with the GOLDSCORE scoring function and a radius of 15 Å, at 0.55.

**Figure 7:**
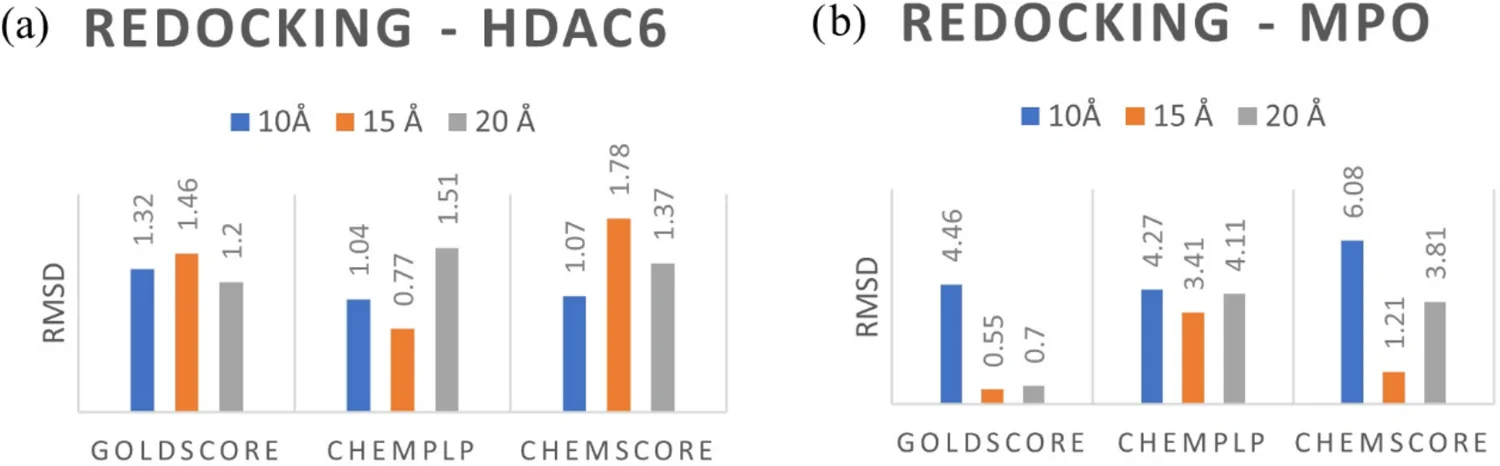
RMSD values obtained by redocking using different scoring functions applied to targets (a) HDAC6 and (b) MPO.

Figure 8 depicts the overlap between the cocrystallized molecules and the best poses obtained from the redocking studies, using the best scoring functions for HDAC6 and MPO.

**Figure 8:**
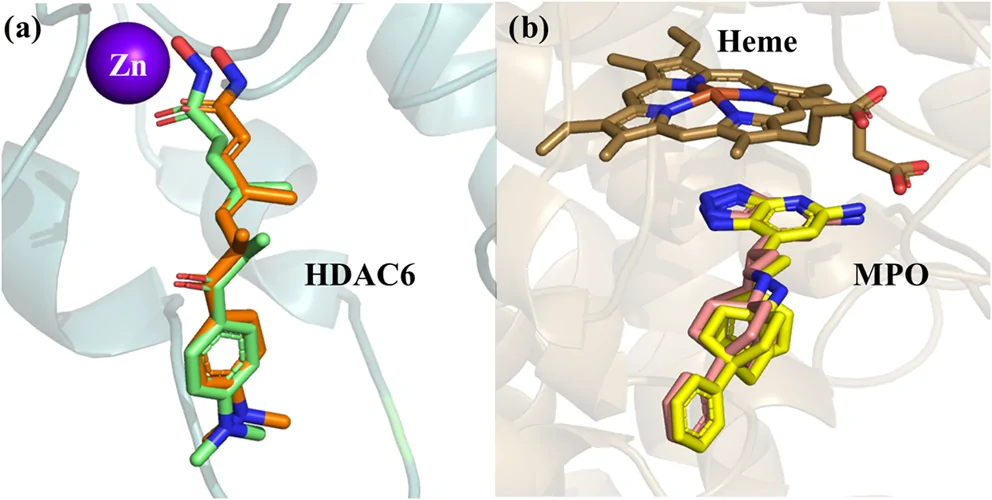
Best RMSD values obtained for the redocking of: (a) HDAC6, with an RMSD of 0.77 using the ChemPLP scoring function and a 15 Å radius; cocrystallized ligand with green carbon atoms and top docking pose in orange; (b) MPO, with an RMSD of 0.55 using the GoldScore scoring function and a 15 Å radius; cocrystallized ligand with yellow carbon atoms and top docking pose in pink.

Based on the best parameters obtained from redocking, molecular docking was performed for all ligands selected in the previous step for each receptor, as well as for reference molecules associated with these ligands. Table S10 (see Supporting Information) contains the scores obtained for all studied ligands, and Figures S1 and S2 show box plot charts comparing the molecular docking scores obtained for the reference compounds and the candidate compounds. Furthermore, Figure 9 shows the score values for all ligands studied for both HDAC6 and MPO, as well as those obtained from reference compounds for these receptors. The candidate ligands showed no statistically significant difference compared to the MPO reference inhibitors (Figure S1a) but differed from those specific for HDAC6 (Figure S1b). When comparing the candidates across the combined reference set, there is no statistical difference between the groups (Figure S2a,b). This behavior reflects the expected affinity trade-off in multitarget design.^25,26,28^ As selective inhibitors are highly optimized for a single cavity, dual molecules tend to exhibit slightly lower scores on one target to accommodate both targets. Finally, beyond the scores, an in-depth analysis of protein–ligand interactions is essential for selecting consistent prototypes and guiding future structural optimization.

**Figure 9:**
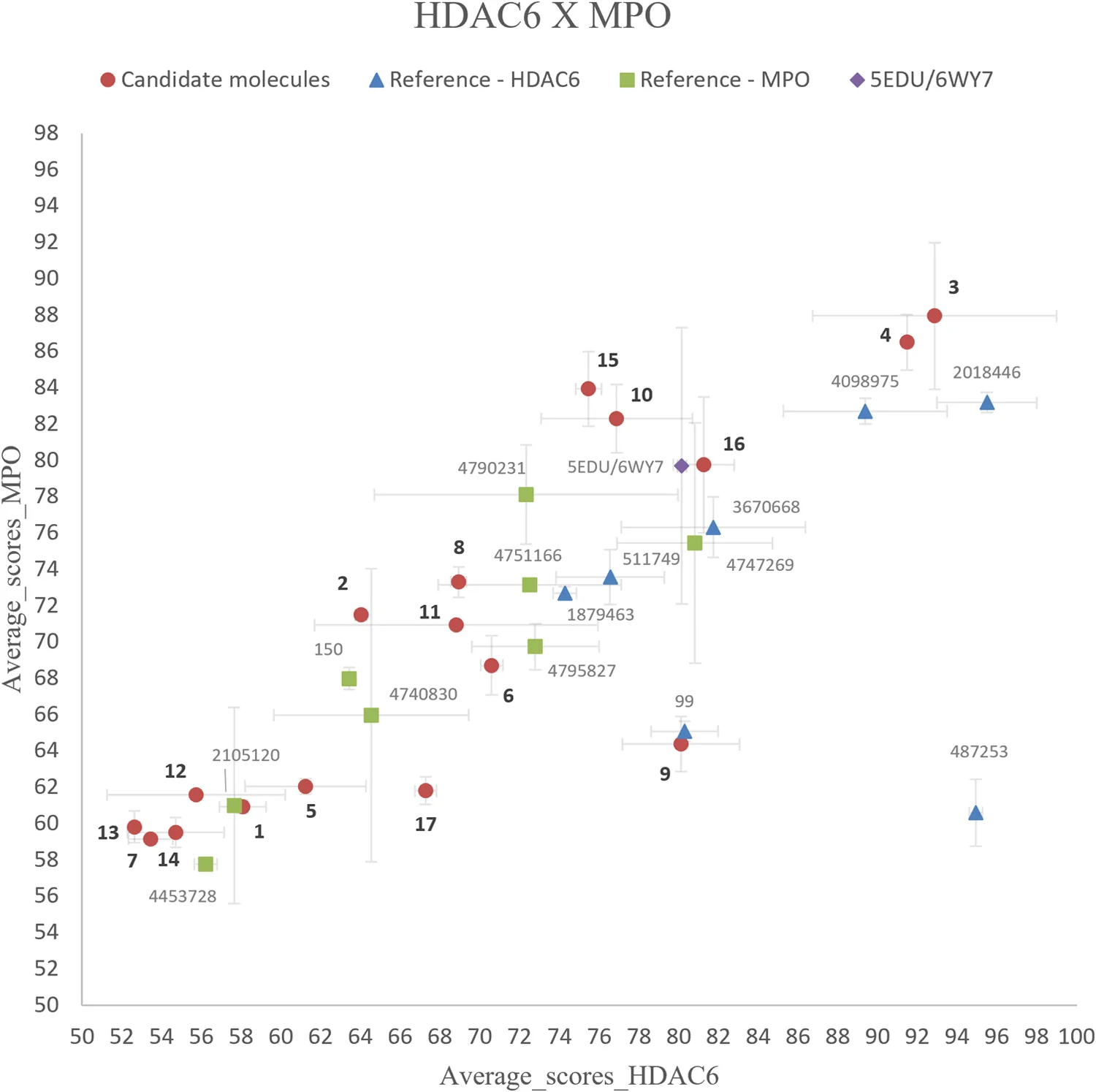
Molecular docking scores between selected molecules and targets HDAC6 and MPO.

For HDAC6, the reference compounds CHEMBL2018446 and CHEMBL487253 had the highest scores, followed by candidates **3** and **4**. Regarding MPO, the highest score was observed for candidates **3**, **4**, and **15**, followed by the reference compounds CHEMBL2018446 and CHEMBL4098975. As shown in the plot, when the candidate compounds that simultaneously yielded the highest scores for both HDAC6 and MPO were compared, eight compounds were identified with scores of above 68. In addition to molecules **3** and **4**, compounds **10**, **15**, and **16** were selected, followed by compounds **6**, **8**, and **11** (Figure 10). Subsequently, the analysis of eight potential ligands was performed to evaluate their putative intermolecular interactions with each protein target.

**Figure 10:**
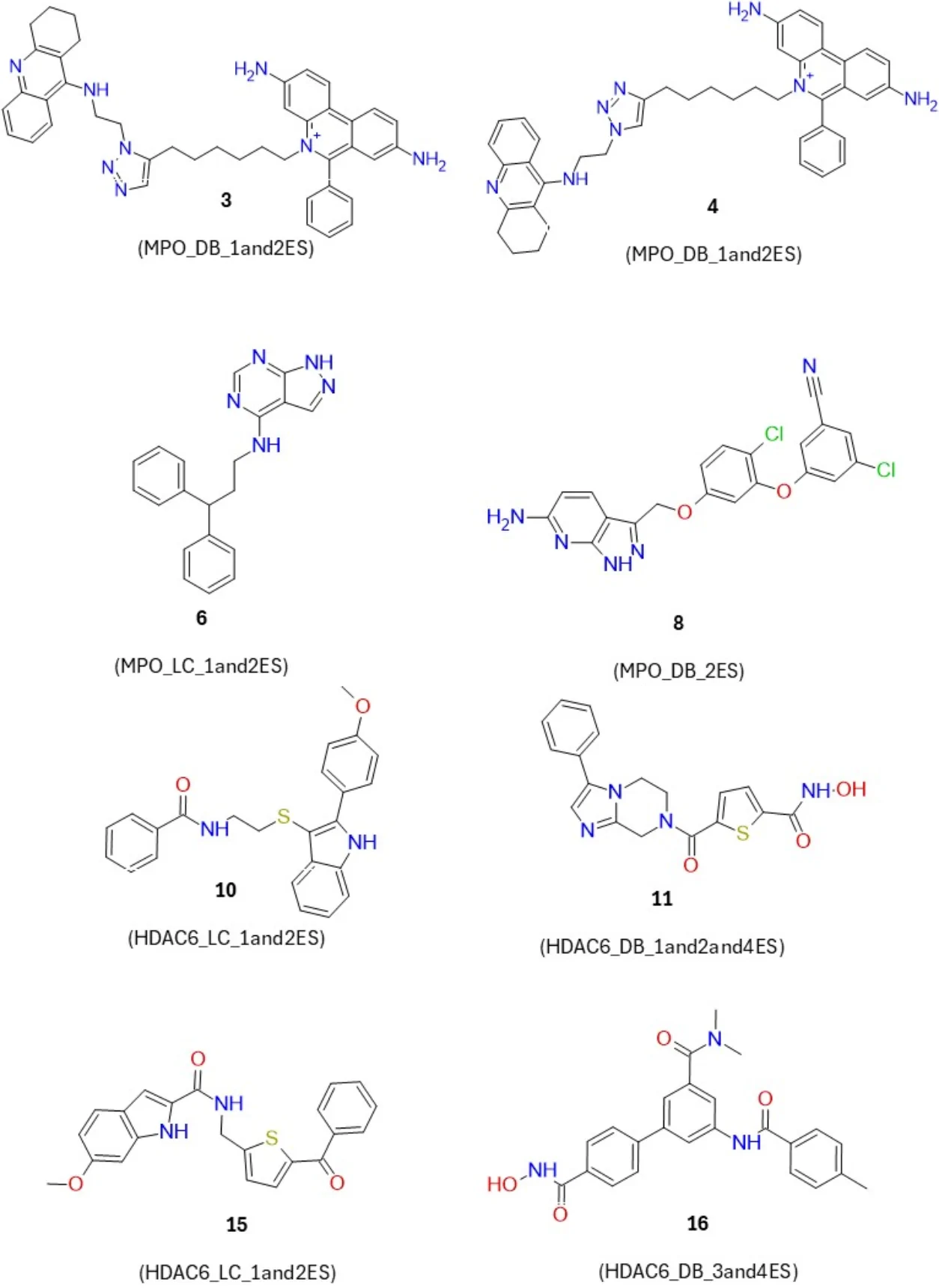
Potential ligands selected from molecular docking (**DB**: DrugBank; **LC**: Life Chemicals; **E**: Strategy) used for the selection of reference compounds in the similarity study.

When analyzing the interactions related to the reference ligand—CHEMBL2018446 with HDAC6, it is possible to observe the coordination of Zn^2+^ with the oxygen atom of the carbonyl group at a bonding distance of 2.18 Å. In addition, it exhibited a total of four hydrogen bonds involving the carbonyl oxygen of Gly619, the ND1 nitrogen atom of the side chain of His611 and the N–H hydrogen of the hydroxamate moiety of CHEMBL2018446; the hydrogen atom HE2 of the imidazole ring of His610, and the hydroxyl oxygen of the hydroxamate; and the hydrogen atom HD1 of His651 with the carbonyl oxygen of the hydroxamate, as well as with the carbonyl oxygen atoms of the amino acid residues Leu749 and Thr678 through carbon–hydrogen bonds, Finally, it interacts with Phe679 via π-cation interactions (Figure 11a). For the interaction of CHEMBL2018446 with MPO (Figure 11b), hydrogen bonding with His95 through the imidazole nitrogen atom (NE2) and Gln91 involving the side chain amide nitrogen (NE2) with the hydroxamate carbonyl oxygen can be observed. Furthermore, it established π–alkyl interactions with Hec601 (HEC refers to the HEME C group), in addition to other π-type interactions involving Arg239 (NH1), Met411 (SD), Leu216 (CD1), Thr238 (O), Phe366, and Cys232 (Figure 11b).

**Figure 11:**
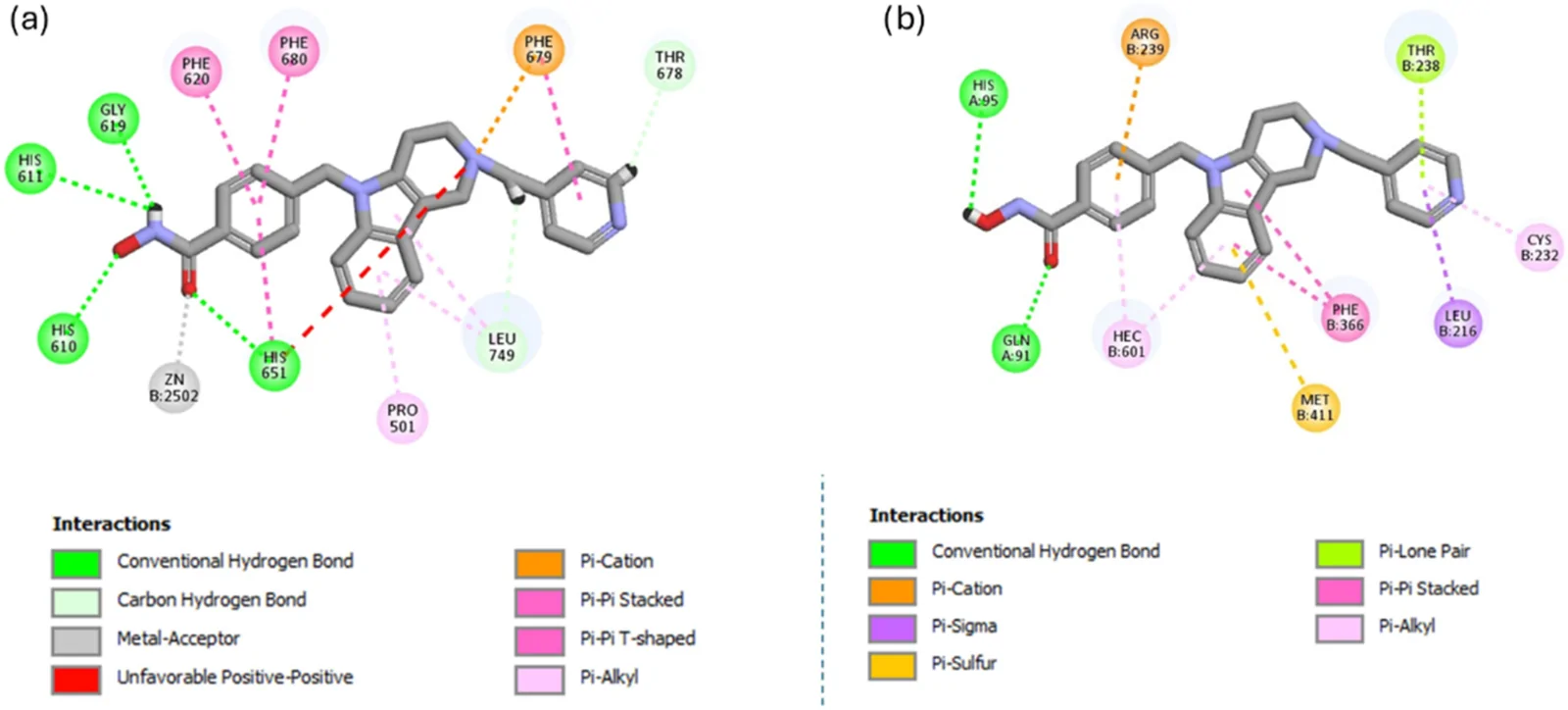
Interactions between the reference molecule CHEMBL2018446 with (a) HDAC6 and (b) MPO.

The molecular interactions of the best ligand poses with HDAC6 are shown in Figure 12. The 3D ligand-HDAC6 complexes can be viewed in Figure S1 (see Supporting Information).

**Figure 12:**
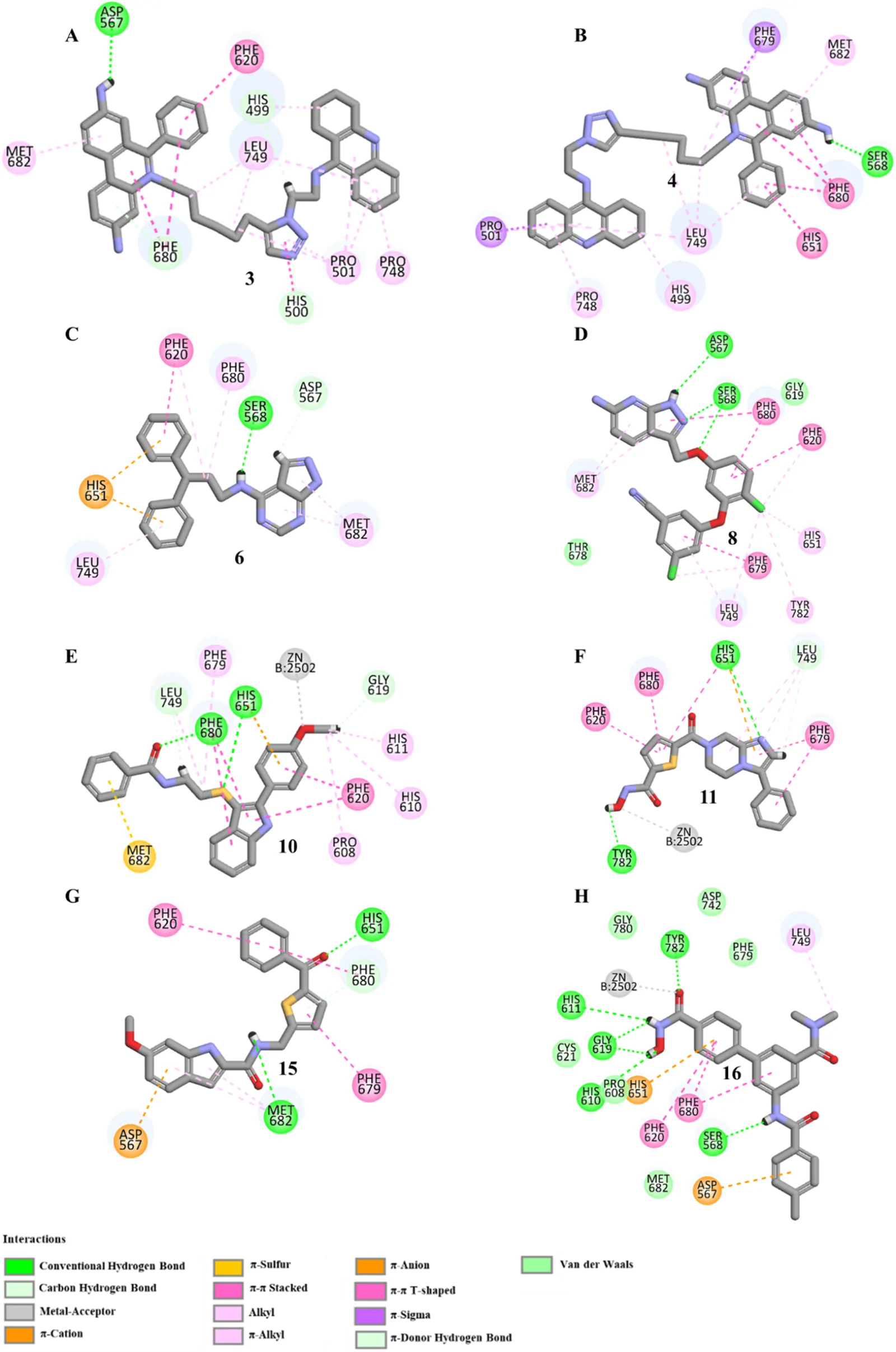
Interactions observed by docking with HDAC6 for molecules (A) **3**; (B) **4**; (C) **6**; (D) **8**; (E) **10**; (F) **11**; (G) **15**; and (H) **16**.

The literature reports several key interactions with amino acid residues of HDAC6, in addition to the interaction with Zn^2+^, involved in HDAC6 inhibition, including hydrogen bonding with His610, His611, and Tyr782; π–π interactions established with Phe620 and Phe680; and nonpolar interactions with Ser568, Gly619, His651 residues.^15,52,53^ Upon analyzing the ligand–target interactions in Discovery studio, it was observed that, among the selected molecules, only **10, 11**, and **16** exhibited interactions with Zn^2+^, with bond distances of 2.67, 2.05, and 2.30 Å, respectively (Figure 12). As shown in Table 4, in addition to interactions with the Zn^2+^ ion, hydrogen bonds were also identified between the ligands and the active-site residues.

**Table 4:** Average Scores and Main Interactions between the Candidate Ligands and the Active-Site Residues of HDAC6

**ligand ID**	**average score**	**no. of H-bonds**	**H-bond_residues**	**H-bond_distances (Å)**	**no. of metal–ligand interactions**	**metal–ligand distances (Å)**	**total strong interactions**
**CHEMBL2018446**	95.49	4	Gly619; His611; His610; His651	1.94; 2.58; 2.20; 2.85	1	2.18	5
**3**	92.86	1	Asp567	2.58	0		1
**4**	91.47	1	Ser568	2.00	0		1
**6**	70.59	1	Ser568	2.11	0		1
**8**	68.93	3	Asp567; Ser568; Ser568;	2.66; 2.08; 2.57	0		3
**10**	76.88	2	Phe680; His651	2.20; 2.61	1	2.67	3
**11**	68.80	2	His651; Tyr782	2.98; 1.78	1	2.05	3
**15**	75.46	2	His651; Met682	1.88; 2.37	0		2
**16**	81.25	6	Tyr782; His611; Gly619; Gly619; His610; Ser568	1.73; 2.71; 1.78; 2.91; 2.61; 1.93	1	2.30	7

In a similar way to the reference compound CHEMBL2018446, molecule **16** established hydrogen bonds between its hydroxamate group and the backbone carbonyl oxygen of Gly619, and with the side chains of His611 and His610, involving, respectively, the ND1 nitrogen atom and the HE2 hydrogen atom of the imidazole ring. Additionally, two other hydrogen bonds were observed between the ligand and the hydroxyl oxygen (OG) of Ser568, as well as with the phenolic hydroxyl group of Tyr782, resulting in a total of six interactions with bond distances shorter than 3.0 Å. These results suggest that molecule **16** exhibits greater inhibitory potential against HDAC6 than the other candidates, making it a strong candidate as an HDAC6 inhibitor. Additionally, molecule **11** exhibited hydrogen-bond interactions between its imidazole ring and His651 (HE2), in addition to a hydrogen bond between its hydroxamate group and the oxygen atom of the hydroxyl of Tyr782. It also performed a carbon–hydrogen interaction with Leu749 and a π-cation interaction with residue His651. Ligand **10**, in turn, exhibited hydrogen-bond interactions between its hydroxamate group and the backbone NH hydrogen atom of Phe680, as well as an additional hydrogen-bond interaction between its sulfur atom and the HE2 hydrogen of the imidazole ring of His651. Furthermore, carbon–hydrogen-bond interactions were observed with Leu749 and Gly619, along with a π–cation interaction with His651. Although molecule **8** forms three hydrogen bonds in total, one with Asp567 (OD1) and two bonds with the hydroxyl hydrogen atom of Ser568, this molecule did not exhibit any interaction with the Zn^2+^ ion, which is considered one of the key interactions associated with inhibitory potential against HDAC6. The best docking poses are shown in Figure S3, and all ligand interactions with HDAC6 target amino acid residues are listed in Table S11, provided in the Supporting Information.

As shown in Figure 13, the surface contours for H-bond donors (pink) and acceptors (green) of molecules **11** and **16** reveal spatial alignment within the HDAC6 active site, facilitating direct coordination with Zn^2+^.

**Figure 13:**
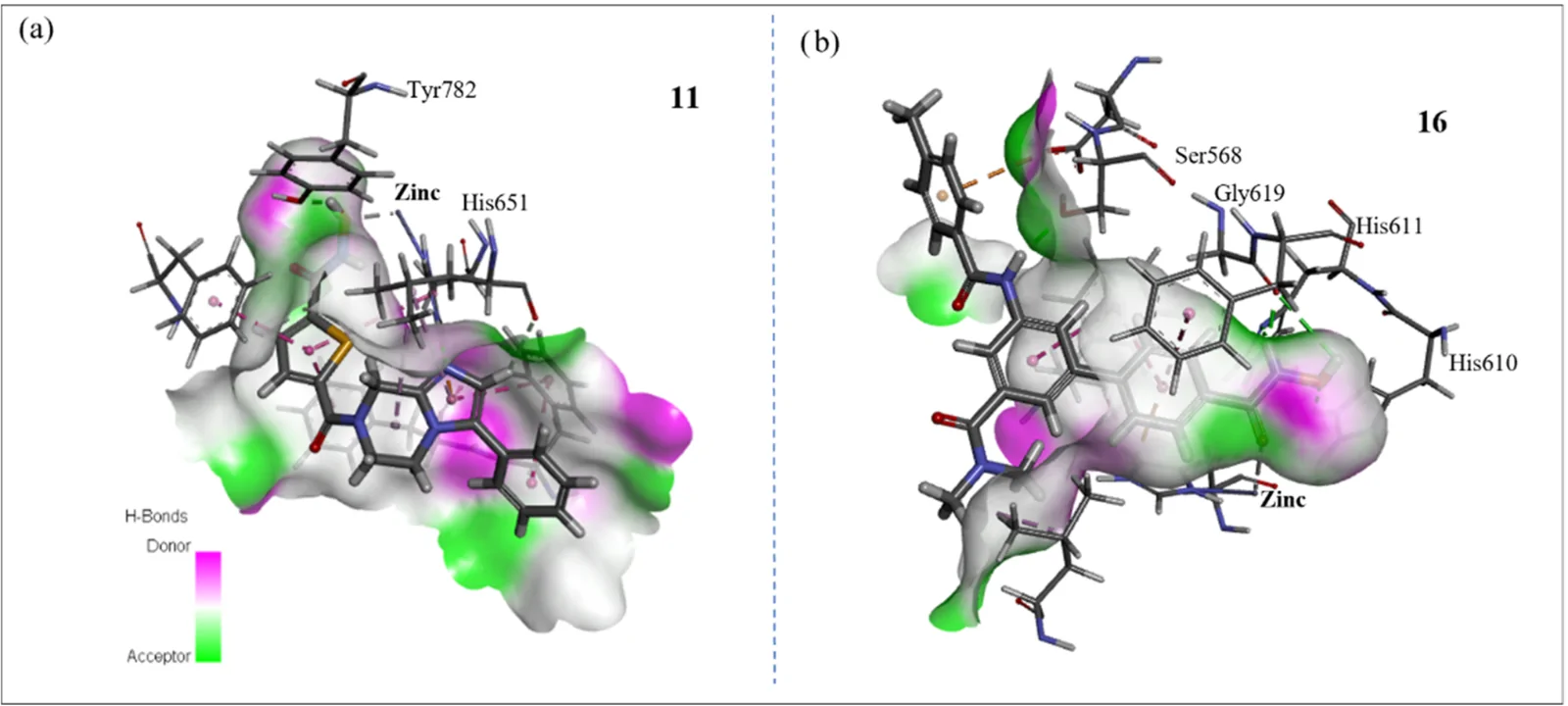
Surface contours for H-bond donors and acceptors of molecules at the Zn^2+^ coordination site of HDAC6 for (a) molecule **11** and (b) molecule **16**.

The molecular interactions of the best ligand poses with MPO are shown in Figure 14, and the 3D ligand-MPO complexes can be viewed in Figure S4 (see Supporting Information). As previously reported in the literature, aromatic molecules tend to bind near the distal cavity of the heme group within MPO, which accommodates a triad of amino acids: His95, Arg239, and Gln91. Additionally, other amino acid residues, such as Phe99, Phe366, and Phe407, are present in this region.^17,18^ When compared to the candidate ligands, hydrogen-bond interactions involving the O2D oxygen atom of the Hec601 group were observed for molecules **8** and **15** (at bond distances of 1.91 and 2.61 Å, respectively) as shown in Table 5. Most of the molecules exhibited hydrogen-bonding interactions with key amino acid residues, including Ser149 through its side chain hydroxyl oxygen atom (OG), Pro101 through its backbone carbonyl oxygen atom (O), Gln91 involving the side chain amide nitrogen (NE2), His95 through the imidazole nitrogen atom (NE2), and Arg239 via terminal guanidinium nitrogen (NH1). In contrast, molecule **10** does not show any significant hydrogen bonding. These interactions suggest a potential stabilization of the ligand within the binding site and highlight the importance of these residues in molecular recognition and binding affinity. Overall, other predominant interactions, such as π-cation, π-anion, and π–π stacking, were observed (Figure 14). Table S12 details the interactions identified between each ligand and the amino acid residues involved in the catalytic site of MPO. The interaction profile obtained through docking suggests that molecule **6** establishes three key interactions, which may indicate a greater ability to accommodate within the active site of MPO. Molecules **3**, **8**, **11**, **15**, **16**, in turn, displayed two hydrogen bonds each, along with other interactions (Figure 14).

**Figure 14:**
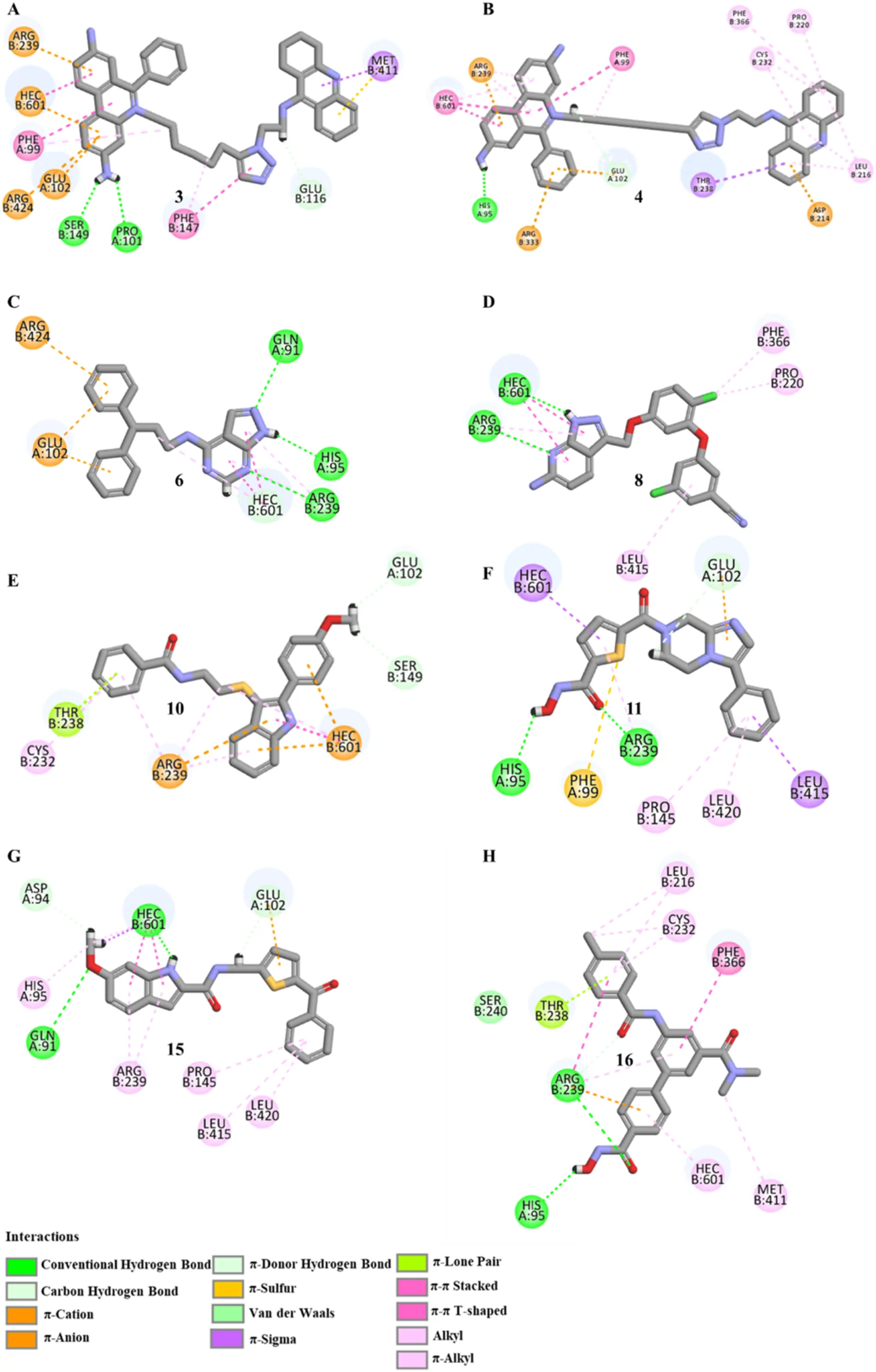
Interactions observed by docking with MPO for molecules (A) **3**; (B) **4**; (C) **6**; (D) **8**; (E) **10**; (F) **11**; (G) **15**; and (H) **16**.

**Table 5:** Main Interactions between the Candidate Ligands and the Active Site of MPO

**ligand ID**	**average score**	**no. of H-bonds**	**H-bond residues/metal**	**H-bond distances (Å)**	**total strong interactions**
**CHEMBL2018446**	83.17	2	His95; Gln91	2.63; 3.18	2
**3**	87.94	2	Ser149; Pro101	2.17; 2.75	2
**4**	86.49	1	His95	3.06	1
**6**	68.70	3	Gln91; His95; Arg239	2.93; 2.62; 2.88	3
**8**	73.30	2	Hec601; Arg239	1.91; 2.75	2
**10**	82.28	0			0
**11**	70.92	2	His95; Arg239	2.38; 3.32	2
**15**	83.93	2	Hec601; Gln91	2.61; 3.31	2
**16**	79.74	2	Arg239; His95	3.28; 2.91	2

The presence of a hydroxamate pharmacophoric group characterizes molecules **10**, **11**, **15**, and **16**. Among them, as shown in Figure 15, molecules **11** and **16** adopt a binding pose in which the oxygen atom of the hydroxamate group is directly oriented toward the Fe^3+^ iron ion of the heme group (Hec601), suggesting metal coordination.

**Figure 15:**
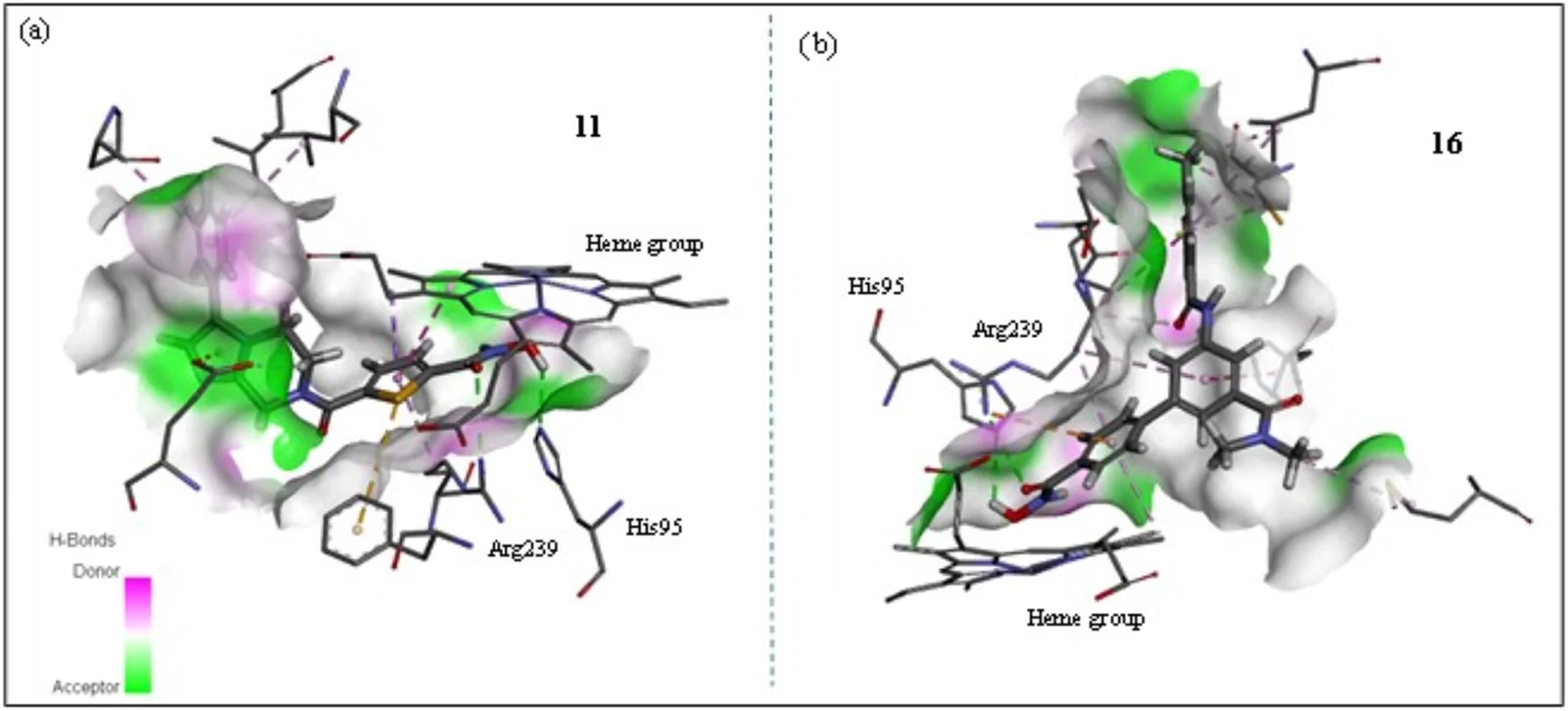
Surface contours for H-bond donors and acceptors at the Hec601 site of MPO for (a) molecule **11** and (b) molecule **16**.

Although proximity to the catalytic metal can, in some chemical classes, favor heme-mediated oxidation, Forbes et al. (2013) established that aromatic hydroxamates constitute a privileged class of potent reversible inhibitors of MPO.^19^ According to crystallographic data reported in the literature, the thermodynamically favorable chelation to Fe^3+^, combined with the steric filling of the access channel by the ligand’s hydrophobic and aromatic moieties, robustly stabilizes the enzyme in its native resting state. This type of interaction can physically block access to hydrogen peroxide, reversibly inhibiting the catalytic cycle before Compound I can form. Thus, the enzyme’s oxidative capacity is suppressed without inducing permanent covalent modifications to the protein structure or generating unwanted radical byproducts. These results, although still preliminary and limited to the computational level, indicate that the candidates exhibit similar molecular recognition patterns, reinforcing the need for additional experimental evaluations to understand better their differences in affinity and inhibitory activity against MPO.

As previously discussed, molecules **10, 11**, and **16** exhibited key interactions relevant to the inhibition of HDAC6, positioning them as potential inhibitors for this target. In contrast, for MPO, molecule **6** exhibits the highest number of hydrogen-bonding interactions. However, other molecules, such as **8**, **11**, **15**, and **16**, also showed significant potential interactions with the amino acids His95, Arg239, and Gln91 in the triad. Finally, molecules **11** and **16** exhibit a favorable bonding conformation, with the oxygen atom of the hydroxamate group positioned directly toward the Fe^3+^ ion of the heme group (Hec601).

Based on this discussion, molecules **11** and **16** emerge as possible dual inhibitors of HDAC6 and MPO, exhibiting, in theory, relevant molecular interactions with the active sites of both targets. Table S13 presents the structural identification (IUPAC, SMILES), and the physicochemical characterization of these two compounds. In short, their theoretical binding profiles suggest potential for application in multitarget therapeutic strategies, as they established key interactions with both targets. Molecule **16** was identified in the DrugBank database using a strategy based on HDAC6 reference compounds (strategies 3 and 4), aimed at expanding structural representativity by combining chemical diversity with inhibitory potential. Additionally, molecule **11** was identified from the DrugBank database through a strategy based on HDAC6 reference compounds (strategies 1, 2, and 4). It is worth noting that the full validation of the predictive hypothesis built from our data requires overcoming significant experimental and pharmacological barriers. One key challenge is the selectivity of HDAC6 over other HDAC isozymes, particularly those of Class I (HDAC1, 2, 3, and 8), since it is well-known that nonselective (*pan*-HDAC) inhibitors frequently cause undesirable side effects, such as hematological, gastrointestinal, or cardiac toxicity.^15,52^ Regarding MPO, challenges stem from the protein’s complex enzymology and ligand selectivity for related heme peroxidases, such as thyroid peroxidase (TPO) and lactoperoxidase (LPO), as inadvertent inhibition of these enzymes may trigger adverse reactions.^17^ Furthermore, the efficacy of many reversible inhibitors is limited in physiological environments by the presence of natural reducing agents, which can reverse inhibition and restore the enzyme to its active state.^18,23^ Clinically, this strategy aims not only to simultaneously modulate multiple targets but also to enhance efficacy and safety.^25^ Multitarget inhibition aims to achieve synergistic effects within the pathophysiological network by simultaneously disrupting multiple disease-related pathways, offering a promising alternative for optimizing the treatment of complex diseases.^26,28^ Nevertheless, translating this hypothesis faces significant future challenges, particularly in optimizing a pharmacokinetic and pharmacotechnical profile that ensures effective delivery of the ligand to distinct biological pathways.^15^ Therefore, this work serves as a proof-of-concept and a structural starting point. Nevertheless, future exploration of the identified hit compounds will require experimental *in vitro* assays and lead optimization to confirm bioactivity and facilitate the development of novel therapies for CVDs.

## 4 Conclusions

In this work, a workflow for chemical space analysis was developed to obtain reference molecules with similar chemical profiles, thereby facilitating subsequent identification of HDAC6 and MPO compounds. On the basis of this approach, a similarity study was conducted between the reference molecules and the database, yielding maximum similarity values above 80% and the selection of 17 molecules for the docking study.

From a detailed analysis of molecular interactions in the best docking poses, compounds **11** and **16** emerged as possible dual inhibitors, standing out for their ability to interact with key amino acid residues from both targets. These compounds exhibited coordination interactions with the Zn^2+^ ion at the active site of HDAC6 as well as interactions with the heme group cavity involving the MPO amino acid triad, which is considered essential for inhibitory activity. Overall, the multistep virtual screening process integrating chemical space analysis, virtual screening, and molecular docking proved to be effective in identifying and prioritizing ligands with multitarget potential. In short, this approach represents a rational and expandable strategy for developing new dual HDAC6 and MPO inhibitors, advancing drug discovery efforts that target cardiovascular diseases.

## Supplementary Material



## Notes

Reference compounds for HDAC6 and MPO selected in the first and third selection strategies; detailed descriptions of KNIME workflow nodes; sensitivity analyses for Euclidean distance thresholds; molecular docking scores, box plot comparisons, best binding poses, and amino acid residue interactions with HDAC6 and MPO; and structural identification and physicochemical characterization of potential dual-target ligands (DOCX)

## Abbreviations

HDAC6: histone deacetylase 6
MPO: myeloperoxidase
CVDs: cardiovascular diseases
GOLD: genetic optimization for ligand docking
MW: molecular weight
nHBDon: number of hydrogen-bond donors
nHBAcc: number of hydrogen-bond acceptors
TopoPSA: topological polar surface area
nRotB: number of rotatable bonds
ALogP: octanol–water partition coefficient (calculated)
PCA: principal component analysis
PC: principal component
MACCS: molecular access system
PDB: Protein Data Bank
IC_50_: half-maximal inhibitory concentration
DB: DrugBank
LC: LifeChemicals
ES: EStrategy
Arg: arginine
Asp: aspartic acid
Cys: cysteine
Glu: glutamic acid
Gln: glutamine
Gly: glycine
His: histidine
Leu: leucine
Met: methionine
Phe: phenylalanine
Pro: proline
Ser: serine
Thr: threonine
Trp: tryptophan
Tyr: tyrosine
Zn: zinc
Fe: iron
HEC: heme C
