# Supplementary material for: Virtual Screening for Dual Inhibitors of HDAC6 and MPO: A Multistep Approach Integrating Chemical Space Analysis and Molecular Docking
Source: ACS Omega. 2026 Oct 16;11(42):64597–612. doi: 10.1021/acsomega.6c04249 (PMC13717808; doi:10.1021/acsomega.6c04249)
Supplement: Supplementary file 1 [file ao6c04249_si_001.docx]

Virtual Screening for Dual Inhibitors of HDAC6 and MPO: A Multi-Step Approach Integrating Chemical Space Analysis and Molecular Docking

*Bárbara M. de Viveiros^a,b^, Camilo H. S. Lima^b^, Nelilma C. Romeiro^a^**

^a^*Laboratório Integrado de Computação Científica – LICC, Instituto Multidisciplinar de Química, Centro Multidisciplinar da Universidade Federal do Rio de Janeiro Macaé, Macaé, RJ, Brazil

^b^Laboratório de Modelagem Molecular, Departamento de Química Orgânica, Instituto de Química, Universidade Federal do Rio de Janeiro, Rio de Janeiro, RJ, Brazil

*nelilmaromeiro@gmail.com

Table S1: Reference compounds for HDAC6 with the lowest IC₅₀ values, according to the first selection strategy.

|  | Nº |  | Molecule | IC_50_ | CHEMBL ID |
| --- | --- | --- | --- | --- | --- |
|  | 1 |  |  | 0.002 nM | CHEMBL4876454 |
|  | 2 |  |  | 0.14 nM | CHEMBL4098975 |
|  | 3 |  |  | 0.17 nM | CHEMBL3912061 |
|  | 4 |  |  | 0.17 nM | CHEMBL4550526 |
|  | 5 |  |  | 0.2 nM | CHEMBL2417782 |
|  | 6 |  |  | 0.2 nM | CHEMBL4243347 |
|  | 7 |  |  | 0.25 nM | CHEMBL4247128 |
|  | 8 |  |  | 0.275 nM | CHEMBL4748025 |
|  | 9 |  |  | 0.29 nM | CHEMBL4104117 |
|  | 10 |  |  | 0.3 nM | CHEMBL4863515 |
|  | 11 |  |  | 0.323 nM | CHEMBL3621294 |
|  | 12 |  |  | 0.33 nM | CHEMBL4126811 |
|  | 13 |  |  | 0.351 nM | CHEMBL4756236 |
|  | 14 |  |  | 0.4  nM | CHEMBL4777296 |
|  | 15 |  |  | 0.4  nM | CHEMBL99 - TRICHOSTATIN |
|  | 16 |  |  | 0.42 nM | CHEMBL4749714 |
|  | 17 |  |  | 0.44 nM | CHEMBL3670668 |
|  | 18 |  |  | 0.46 nM | CHEMBL2018447 |
|  | 19 |  |  | 0.5 nM | CHEMBL3759794 |
|  | 20 |  |  | 0.5 nM | CHEMBL4177129 |
|  | 21 |  |  | 0.5 nM | CHEMBL4536605 |
|  | 22 |  |  | 0.58 nM | CHEMBL2018446 |
|  | 23 |  |  | 0.59 nM | CHEMBL4751524 |
|  | 24 |  |  | 0.6 nM | CHEMBL3759982 |
|  | 25 |  |  | 0.60 nM | CHEMBL4777764 |
|  | 26 |  |  | 0.61 nM | CHEMBL4531853 |
|  | 27 |  |  | 0.64 nM | CHEMBL4762877 |
|  | 28 |  |  | 0.64 nM | CHEMBL4170617 |
|  | 29 |  |  | 0.65 nM | CHEMBL4160632 |
|  | 30 |  |  | 0.7 nM | CHEMBL3670669 |

Table S2: Reference compounds for MPO with the lowest IC₅₀ values, according to the first selection strategy.

|  | Nº |  | Molecule | IC_50_ | CHEMBL ID |
| --- | --- | --- | --- | --- | --- |
|  | 1 |  |  | 1 nM | CHEMBL4747269 |
|  | 2 |  |  | 1 nM | CHEMBL4790231 |
|  | 3 |  |  | 2 nM | CHEMBL4763667 |
|  | 4 |  |  | 2.9 nM | CHEMBL4752248 |
|  | 5 |  |  | 3 nM | CHEMBL2347170 |
|  | 6 |  |  | 4 nM | CHEMBL2347168 |
|  | 7 |  |  | 4.6 nM | CHEMBL4548537 |
|  | 8 |  |  | 5 nM | CHEMBL1630742 |
|  | 9 |  |  | 5 nM | CHEMBL2347177 |
|  | 10 |  |  | 5 nM | CHEMBL4068067 |
|  | 11 |  |  | 5 nM | CHEMBL4740830 |
|  | 12 |  |  | 5 nM | CHEMBL4861370 |
|  | 13 |  |  | 6 nM | CHEMBL4782307 |
|  | 14 |  |  | 6.7  nM | CHEMBL4453728 |
|  | 15 |  |  | 7  nM | CHEMBL4851535 |
|  | 16 |  |  | 8 nM | CHEMBL1630741 |
|  | 17 |  |  | 16 nM | CHEMBL4776850 |
|  | 18 |  |  | 8 nM | CHEMBL4878307 |
|  | 19 |  |  | 8.5 nM | CHEMBL4594116 |
|  | 20 |  |  | 8.7 nM | CHEMBL4482878 |
|  | 21 |  |  | 9.3 nM | CHEMBL4751166 |
|  | 22 |  |  | 10 nM | CHEMBL2347166 |
|  | 23 |  |  | 10 nM | CHEMBL2347167 |
|  | 24 |  |  | 11 nM | CHEMBL2347176 |
|  | 25 |  |  | 12 nM | CHEMBL4278946 |
|  | 26 |  |  | 12 nM | CHEMBL4531688 |
|  | 27 |  |  | 13 nM | CHEMBL2347165 |
|  | 28 |  |  | 14 nM | CHEMBL2346670 |
|  | 29 |  |  | 14 nM | CHEMBL4877383 |
|  | 30 |  |  | 15 nM | CHEMBL4795827 |

Table S3: Reference compounds for HDAC6 with the lowest IC₅₀ values, according to the third selection strategy.

|  | Nº |  | Molecule | IC_50_ | CHEMBL ID |
| --- | --- | --- | --- | --- | --- |
|  | 1 |  |  | 0.002 nM | CHEMBL4876454 |
|  | 2 |  |  | 0.14 nM | CHEMBL4098975 |
|  | 3 |  |  | 0.17 nM | CHEMBL3912061 |
|  | 4 |  |  | 0.17 nM | CHEMBL4550526 |
|  | 5 |  |  | 0.2 nM | CHEMBL2417782 |
|  | 6 |  |  | 0.2 nM | CHEMBL4243347 |
|  | 7 |  |  | 0.25 nM | CHEMBL4247128 |
|  | 8 |  |  | 0.275 nM | CHEMBL4748025 |
|  | 9 |  |  | 0.29 nM | CHEMBL4104117 |
|  | 10 |  |  | 0.3 nM | CHEMBL4863515 |
|  | 11 |  |  | 0.323 nM | CHEMBL3621294 |
|  | 12 |  |  | 0.33 nM | CHEMBL4126811 |
|  | 13 |  |  | 0.351 nM | CHEMBL4756236 |
|  | 14 |  |  | 0.4  nM | CHEMBL4777296 |
|  | 15 |  |  | 0.4  nM | CHEMBL99 - TRICHOSTATIN |
|  | 16 |  |  | 1.4 nM | CHEMBL98 -  VORINOSTAT |
|  | 17 |  |  | 1.6 nM | CHEMBL2179618 - NEXTURASTAT A |
|  | 18 |  |  | 1.9 nM | CHEMBL2018302 - TUBASTATIN A |
|  | 19 |  |  | 2 nM | CHEMBL483254 - PANOBINOSTAT |
|  | 20 |  |  | 2 nM | CHEMBL1879463 - DACTOLISIB |
|  | 21 |  |  | 2.21 nM | CHEMBL3693786 -  CITARINOSTAT |
|  | 22 |  |  | 2.59 nM | CHEMBL2364628 - RICOLINOSTAT |
|  | 23 |  |  | 2.9 nM | CHEMBL96051 - SCRIPTAID |
|  | 24 |  |  | 4 nM | CHEMBL356769 - TUBACIN |
|  | 25 |  |  | 5.93 nM | CHEMBL356066 - DACINOSTAT |
|  | 26 |  |  | 6 nM | CHEMBL487253 - BENDAMUSTINE |
|  | 27 |  |  | 8 nM | CHEMBL3622533 - FIMEPINOSTAT |
|  | 28 |  |  | 15 nM | CHEMBL1789941 - RUXOLITINIB |
|  | 29 |  |  | 9.85 nM | CHEMBL408513 -  BELINOSTAT |
|  | 30 |  |  | 10 nM | CHEMBL27759 -  ENTINOSTAT |

Table S4: Reference compounds for MPO with the lowest IC₅₀ values, according to the third selection strategy.

|  | Nº |  | Molecule | IC_50_ | CHEMBL ID |
| --- | --- | --- | --- | --- | --- |
|  | 1 |  |  | 1 nM | CHEMBL4747269 |
|  | 2 |  |  | 1 nM | CHEMBL4790231 |
|  | 3 |  |  | 2 nM | CHEMBL4763667 |
|  | 4 |  |  | 2.9 nM | CHEMBL4752248 |
|  | 5 |  |  | 3 nM | CHEMBL2347170 |
|  | 6 |  |  | 4 nM | CHEMBL2347168 |
|  | 7 |  |  | 4.6 nM | CHEMBL4548537 |
|  | 8 |  |  | 5 nM | CHEMBL1630742 |
|  | 9 |  |  | 5 nM | CHEMBL2347177 |
|  | 10 |  |  | 5 nM | CHEMBL4068067 |
|  | 11 |  |  | 5 nM | CHEMBL4740830 |
|  | 12 |  |  | 5 nM | CHEMBL4861370 |
|  | 13 |  |  | 6 nM | CHEMBL4782307 |
|  | 14 |  |  | 6.7  nM | CHEMBL4453728 |
|  | 15 |  |  | 7  nM | CHEMBL4851535 |
|  | 16 |  |  | 20 nM | CHEMBL490 - PAROXETINE |
|  | 17 |  |  | 350 nM | CHEMBL86 - METOCLOPRAMIDE |
|  | 18 |  |  | 420 nM | CHEMBL1043 - DAPSONE |
|  | 19 |  |  | 460 nM | CHEMBL2105120 - FTIVAZIDE |
|  | 20 |  |  | 630 nM | CHEMBL4297594 - VERDIPERSTAT |
|  | 21 |  |  | 770 nM | CHEMBL6640 - TRYPTAMINE |
|  | 22 |  |  | 900 nM | CHEMBL276832 - HYDRALAZINE |
|  | 23 |  |  | 980 nM | CHEMBL686 – MEFENAMIC ACID |
|  | 24 |  |  | 1150 nM | CHEMBL50 - QUERCETIN |
|  | 25 |  |  | 1620 nM | CHEMBL1945275 - N'-(4-DIMETHYLAMINOBENZYLIDENE)ISONICOTINOHYDRAZIDE |
|  | 26 |  |  | 1800 nM | CHEMBL23588 - FLUFENAMIC ACID |
|  | 27 |  |  | 1900 nM | CHEMBL3633460 -  PF-06282999 |
|  | 28 |  |  | 2100 nM | CHEMBL56367 -  NIMESULIDE |
|  | 29 |  |  | 2250 nM | CHEMBL54976 - TRYPTOPHAN |
|  | 30 |  |  | 2240 nM | CHEMBL150 - KAEMPFEROL |

Table S5: Detailed description of each node used in the space chemical study workflow developed in the KNIME platform.

| Node | Function |
| --- | --- |
| Table Creator | Manually creates a data table for input (e.g., SMILES, ID). |
| Molecule to CDK | Converts SMILES molecules to the CDK format and displays the 2D structure. |
| Molecular Properties | Calculates molecular properties such as logP, mass, TPSA, etc. |
| Split Collection Column | Splits collection (list) columns into individual columns. Used here for AlogP. |
| Column Rename | Renames columns to shorter or standardized names. |
| Column Filter | Removes unwanted columns, keeping only those needed. |
| Normalizer | Normalizes numerical values. Z-Score normalization (Gaussian) was used. |
| PCA Compute | Performs Principal Component Analysis (PCA) on molecular descriptors. |
| PCA Apply | Applies the trained PCA model to new data. Reduced to 2 dimensions with 77.3% of variance retained. |
| Math Formula | Creates new columns with custom mathematical expressions. |
| Excel Writer | Exports the final data to an Excel (.xlsx) file. |
| Image Writer | Saves images generated in the workflow. |
| Color Manager | Assigns colors to variables in visualizations (e.g., color by receptor type). |
| Scatter Plot | Creates a scatter plot (e.g., PC1 vs. PC2) to visualize the chemical space. |
| Row Splitter | Splits the dataset into two groups based on a condition. |
| Cross Joiner | Performs a Cartesian product between two tables (cross comparisons). Used to map HDAC6 and MPO data. |
| Box Plot | Creates box plots for analyzing the distribution of molecular properties. |

Table S6: Detailed description of each node used in the similarity study workflow developed in the KNIME platform.

| Node | Function |
| --- | --- |
| Table Creator | Manually creates a table with input data (e.g., SMILES, ID). |
| Fingerprints | Generates molecular fingerprints (e.g., MACCS) used for similarity calculations. |
| RDKit Remove Hs | Removes hydrogens from a molecule. |
| Row Filter | Filters rows based on established conditions (*e.g*., exclusion of missing values). |
| RDKit Salt Stripper | Removes salts from molecules. |
| RDKit structure Normalizer | Normalizes molecular structures *(e.g*., fragments, tautomers). |
| RDKit Canon SMILES | Generates canonical SMILES for molecules. Standardizes structures. |
| Fingerprint Similarity | Calculates the similarity index between fingerprints (e.g., Tanimoto coefficient). |
| Ungroup | Expands cells that contain multiple individual rows. |
| RowID | Assigns or redefines unique identifiers for each row (Tanimoto coefficient between reference molecule and database molecule). |
| Cell Splitter | Splits the content of text cells into multiple columns using a delimiter. |
| String Manipulation | Performs text operations (*e.g*., replace, extract, concatenate). |
| Joiner | Joins two tables. |
| Table Manipulator | Table editing tool. |
| Column Filter | Removes unwanted columns. |
| Excel Writer | Exports final data to an Excel file (.xlsx). |

**Table S7**: Sensitivity analysis demonstrating the impact of varying Euclidean distance threshold on molecule retention for the HDAC6 and MPO targets in the second strategy.

| Number of molecules_MPO | Number of molecules_HDAC6 | Limit (Euclidean distance) | MPO recovery (%) | HDAC6 recovery (%) |
| --- | --- | --- | --- | --- |
| 7 | 4 | 0.30 | 23.33 | 13.33 |
| 13 | 5 | 0.40 | 43.33 | 16.67 |
| 15 | 6 | 0.50 | 50.00 | 20.00 |
| 18 | 9 | 0.60 | 60.00 | 30.00 |
| 18 | 12 | 0.70 | 60.00 | 40.00 |
| 19 | 13 | 0.80 | 63.33 | 43.33 |
| 25 | 17 | 0.90 | 83.33 | 56.67 |
| 27 | 17 | 1.00 | 90.00 | 56.67 |
| 27 | 18 | 1.10 | 90.00 | 60.00 |
| 27 | 18 | 1.20 | 90.00 | 60.00 |
| 27 | 18 | 1.30 | 90.00 | 60.00 |
| 27 | 20 | 1.40 | 90.00 | 66.67 |
| 27 | 20 | 1.50 | 90.00 | 66.67 |
| 27 | 21 | 1.60 | 90.00 | 70.00 |
| 29 | 21 | 1.70 | 96.67 | 70.00 |
| 30 | 22 | 1.80 | 100.00 | 73.33 |
| 30 | 22 | 1.90 | 100.00 | 73.33 |
| 30 | 23 | 2.00 | 100.00 | 76.67 |
| 30 | 24 | 2.10 | 100.00 | 80.00 |
| 30 | 24 | 2.20 | 100.00 | 80.00 |
| 30 | 24 | 2.30 | 100.00 | 80.00 |
| 30 | 25 | 2.40 | 100.00 | 83.33 |
| 30 | 25 | 2.50 | 100.00 | 83.33 |
| 30 | 26 | 2.60 | 100.00 | 86.67 |
| 30 | 26 | 2.70 | 100.00 | 86.67 |
| 30 | 26 | 2.80 | 100.00 | 86.67 |
| 30 | 26 | 2.90 | 100.00 | 86.67 |
| 30 | 26 | 3.00 | 100.00 | 86.67 |
| 30 | 27 | 3.10 | 100.00 | 90.00 |
| 30 | 27 | 3.20 | 100.00 | 90.00 |
| 30 | 27 | 3.30 | 100.00 | 90.00 |
| 30 | 28 | 3.40 | 100.00 | 93.33 |
| 30 | 28 | 3.50 | 100.00 | 93.33 |
| 30 | 28 | 3.60 | 100.00 | 93.33 |
| 30 | 29 | 3.70 | 100.00 | 96.67 |
| 30 | 29 | 3.80 | 100.00 | 96.67 |
| 30 | 30 | 3.90 | 100.00 | 100.00 |
| 30 | 30 | 4.00 | 100.00 | 100.00 |
| 30 | 30 | 4.10 | 100.00 | 100.00 |
| 30 | 30 | 4.20 | 100.00 | 100.00 |
| 30 | 30 | 4.30 | 100.00 | 100.00 |
| 30 | 30 | 4.40 | 100.00 | 100.00 |
| 30 | 30 | 4.50 | 100.00 | 100.00 |
| 30 | 30 | 4.60 | 100.00 | 100.00 |
| 30 | 30 | 4.70 | 100.00 | 100.00 |
| 30 | 30 | 4.80 | 100.00 | 100.00 |
| 30 | 30 | 4.90 | 100.00 | 100.00 |
| 30 | 30 | 5.00 | 100.00 | 100.00 |
| 30 | 30 | 5.10 | 100.00 | 100.00 |
| 30 | 30 | 5.20 | 100.00 | 100.00 |
| 30 | 30 | 5.30 | 100.00 | 100.00 |
| 30 | 30 | 5.40 | 100.00 | 100.00 |
| 30 | 30 | 5.50 | 100.00 | 100.00 |
| 30 | 30 | 5.60 | 100.00 | 100.00 |
| 30 | 30 | 5.70 | 100.00 | 100.00 |
| 30 | 30 | 5.80 | 100.00 | 100.00 |
| 30 | 30 | 5.90 | 100.00 | 100.00 |
| 30 | 30 | 6.00 | 100.00 | 100.00 |
| 30 | 30 | 6.10 | 100.00 | 100.00 |
| 30 | 30 | 6.20 | 100.00 | 100.00 |
| 30 | 30 | 6.30 | 100.00 | 100.00 |
| 30 | 30 | 6.40 | 100.00 | 100.00 |
| 30 | 30 | 6.50 | 100.00 | 100.00 |
| 30 | 30 | 6.60 | 100.00 | 100.00 |
| 30 | 30 | 6.70 | 100.00 | 100.00 |
| 30 | 30 | 6.80 | 100.00 | 100.00 |
| 30 | 30 | 6.90 | 100.00 | 100.00 |
| 30 | 30 | 7.00 | 100.00 | 100.00 |
| 30 | 30 | 7.10 | 100.00 | 100.00 |
| 30 | 30 | 7.20 | 100.00 | 100.00 |
| 30 | 30 | 7.30 | 100.00 | 100.00 |
| 30 | 30 | 7.40 | 100.00 | 100.00 |
| 30 | 30 | 7.50 | 100.00 | 100.00 |
| 30 | 30 | 7.60 | 100.00 | 100.00 |
| 30 | 30 | 7.70 | 100.00 | 100.00 |
| 30 | 30 | 7.80 | 100.00 | 100.00 |
| 30 | 30 | 7.90 | 100.00 | 100.00 |
| 30 | 30 | 8.00 | 100.00 | 100.00 |
| 30 | 30 | 8.10 | 100.00 | 100.00 |
| 30 | 30 | 8.20 | 100.00 | 100.00 |

**Table S8**: Sensitivity analysis demonstrating the impact of varying Euclidean distance threshold on molecule retention for the HDAC6 and MPO targets in the fourth strategy.

| Number of molecules_MPO | Number of molecules_HDAC6 | Limit (Euclidean distance) | MPO recovery (%) | HDAC6 recovery (%) |
| --- | --- | --- | --- | --- |
| 1 | 1 | 0.1 | 3.33 | 3.33 |
| 5 | 4 | 0.2 | 16.67 | 13.33 |
| 7 | 8 | 0.3 | 23.33 | 26.67 |
| 8 | 9 | 0.4 | 26.67 | 30.00 |
| 11 | 12 | 0.5 | 36.67 | 40.00 |
| 12 | 15 | 0.6 | 40.00 | 50.00 |
| 16 | 15 | 0.7 | 53.33 | 50.00 |
| 20 | 16 | 0.8 | 66.67 | 53.33 |
| 22 | 17 | 0.9 | 73.33 | 56.67 |
| 24 | 17 | 1 | 80.00 | 56.67 |
| 26 | 19 | 1.1 | 86.67 | 63.33 |
| 27 | 19 | 1.2 | 90.00 | 63.33 |
| 28 | 20 | 1.3 | 93.33 | 66.67 |
| 28 | 20 | 1.4 | 93.33 | 66.67 |
| 29 | 20 | 1.5 | 96.67 | 66.67 |
| 29 | 20 | 1.6 | 96.67 | 66.67 |
| 29 | 20 | 1.7 | 96.67 | 66.67 |
| 30 | 20 | 1.8 | 100.00 | 66.67 |
| 30 | 21 | 1.9 | 100.00 | 70.00 |
| 30 | 21 | 2 | 100.00 | 70.00 |
| 30 | 23 | 2.1 | 100.00 | 76.67 |
| 30 | 25 | 2.2 | 100.00 | 83.33 |
| 30 | 25 | 2.3 | 100.00 | 83.33 |
| 30 | 26 | 2.4 | 100.00 | 86.67 |
| 30 | 26 | 2.5 | 100.00 | 86.67 |
| 30 | 26 | 2.6 | 100.00 | 86.67 |
| 30 | 26 | 2.7 | 100.00 | 86.67 |
| 30 | 26 | 2.8 | 100.00 | 86.67 |
| 30 | 26 | 2.9 | 100.00 | 86.67 |
| 30 | 26 | 3 | 100.00 | 86.67 |
| 30 | 26 | 3.1 | 100.00 | 86.67 |
| 30 | 26 | 3.2 | 100.00 | 86.67 |
| 30 | 27 | 3.3 | 100.00 | 90.00 |
| 30 | 27 | 3.4 | 100.00 | 90.00 |
| 30 | 27 | 3.5 | 100.00 | 90.00 |
| 30 | 27 | 3.6 | 100.00 | 90.00 |
| 30 | 28 | 3.7 | 100.00 | 93.33 |
| 30 | 28 | 3.8 | 100.00 | 93.33 |
| 30 | 29 | 3.9 | 100.00 | 96.67 |
| 30 | 30 | 4 | 100.00 | 100.00 |
| 30 | 30 | 4.1 | 100.00 | 100.00 |
| 30 | 30 | 4.2 | 100.00 | 100.00 |
| 30 | 30 | 4.3 | 100.00 | 100.00 |
| 30 | 30 | 4.4 | 100.00 | 100.00 |
| 30 | 30 | 4.5 | 100.00 | 100.00 |
| 30 | 30 | 4.6 | 100.00 | 100.00 |
| 30 | 30 | 4.7 | 100.00 | 100.00 |
| 30 | 30 | 4.8 | 100.00 | 100.00 |
| 30 | 30 | 4.9 | 100.00 | 100.00 |
| 30 | 30 | 5 | 100.00 | 100.00 |
| 30 | 30 | 5.1 | 100.00 | 100.00 |
| 30 | 30 | 5.2 | 100.00 | 100.00 |
| 30 | 30 | 5.3 | 100.00 | 100.00 |
| 30 | 30 | 5.4 | 100.00 | 100.00 |
| 30 | 30 | 5.5 | 100.00 | 100.00 |
| 30 | 30 | 5.6 | 100.00 | 100.00 |
| 30 | 30 | 5.7 | 100.00 | 100.00 |
| 30 | 30 | 5.8 | 100.00 | 100.00 |
| 30 | 30 | 5.9 | 100.00 | 100.00 |
| 30 | 30 | 6 | 100.00 | 100.00 |
| 30 | 30 | 6.1 | 100.00 | 100.00 |
| 30 | 30 | 6.2 | 100.00 | 100.00 |
| 30 | 30 | 6.3 | 100.00 | 100.00 |
| 30 | 30 | 6.4 | 100.00 | 100.00 |
| 30 | 30 | 6.5 | 100.00 | 100.00 |
| 30 | 30 | 6.6 | 100.00 | 100.00 |
| 30 | 30 | 6.7 | 100.00 | 100.00 |
| 30 | 30 | 6.8 | 100.00 | 100.00 |
| 30 | 30 | 6.9 | 100.00 | 100.00 |
| 30 | 30 | 7 | 100.00 | 100.00 |
| 30 | 30 | 7.1 | 100.00 | 100.00 |
| 30 | 30 | 7.2 | 100.00 | 100.00 |
| 30 | 30 | 7.3 | 100.00 | 100.00 |
| 30 | 30 | 7.4 | 100.00 | 100.00 |
| 30 | 30 | 7.5 | 100.00 | 100.00 |
| 30 | 30 | 7.6 | 100.00 | 100.00 |
| 30 | 30 | 7.7 | 100.00 | 100.00 |
| 30 | 30 | 7.8 | 100.00 | 100.00 |
| 30 | 30 | 7.9 | 100.00 | 100.00 |
| 30 | 30 | 8 | 100.00 | 100.00 |
| 30 | 30 | 8.1 | 100.00 | 100.00 |
| 30 | 30 | 8.2 | 100.00 | 100.00 |

Table S9: Molecules selected for molecular docking after similarity studies.

| **Nº** | **NOMENCLATURE** | **SMILES** |  | **SIMILARITY**  **(%)** |
| --- | --- | --- | --- | --- |
| 1 | 1  (MPO_LC_3and4ES) |  |  | 98% |
| 2 | 2  (MPO_DB_4ES) |  |  | 97% |
| 3 | 3  (MPO_DB_1and2ES) |  |  | 89% |
| 4 | 4  (MPO_DB_1and2ES) |  |  | 89% |
| 5 | 5  (MPO_LC_1ES) |  |  | 89% |
| 6 | 6  (MPO_LC_1and2ES) |  |  | 89% |
| 7 | 7  (MPO_DB_2ES) |  |  | 88% |
| 8 | 8  (MPO_DB_2ES) |  |  | 87% |
| 9 | 9  (HDAC6_LC_1and2ES) |  |  | 86% |
| 10 | 10  (HDAC6_LC_1and2ES) |  |  | 84% |
| 11 | 11 (HDAC6_DB_1and2and4ES) |  |  | 84% |
| 12 | 12  (HDAC6_LC_3ES) |  |  | 84% |
| 13 | 13  (HDAC6_LC_3ES) |  |  | 84% |
| 14 | 14  (HDAC6_LC_1and2ES) |  |  | 83% |
| 15 | 15  (HDAC6_LC_1and2ES) |  |  | 83% |
| 16 | 16  (HDAC6_DB_3and4ES) |  |  | 82% |
| 17 | 17  (HDAC6_LC_4ES) |  |  | 81% |

Table S10: Docking scores and corresponding standard deviations obtained for all ligands in the molecular docking study.

| Ligand | HDAC6 | MPO |
| --- | --- | --- |
| 1 | 58.07 ± 1.18 | 60.92 ± 0.38 |
| 2 | 64.02 ± 0.29 | 71.48 ± 0.24 |
| 3 | 92.86 ± 6.14 | 87.94 ± 4.02 |
| 4 | 91.47 ± 0.24 | 86.49 ± 1.54 |
| 5 | 61.23 ± 3.04 | 62.05 ± 0.43 |
| 6 | 70.59 ± 0.55 | 68.70 ± 1.63 |
| 7 | 53.44 ± 1.11 | 59.14 ± 0.13 |
| 8 | 68.93 ± 0.39 | 73.30 ± 0.84 |
| 9 | 80.11 ± 2.95 | 64.37 ± 1.51 |
| 10 | 76.88 ± 3.79 | 82.28 ± 1.89 |
| 11 | 68.80 ± 7.12 | 70.92 ± 0.19 |
| 12 | 55.74 ± 4.49 | 61.58 ± 0.27 |
| 13 | 52.63 ± 0.27 | 59.81 ± 0.87 |
| 14 | 54.71 ± 2.45 | 59.51 ± 0.83 |
| 15 | 75.46 ± 0.65 | 83.93 ± 2.06 |
| 16 | 81.25 ± 1.55 | 79.74 ± 3.73 |
| 17 | 67.28 ± 0.53 | 61.80 ± 0.75 |
| LIGAND 5ED5/6WY7 | 80.16 ± 0.22 | 79.93 ± 7.40 |
| CHEMBL2105120 (MPO) | 57.64 ± 0.24 | 60.98 ± 5.41 |
| CHEMBL150  (MPO) | 63.43 ± 0.28 | 67.97 ± 0.61 |
| CHEMBL4795827 (MPO) | 72.79 ± 3.19 | 69.73 ± 1.26 |
| CHEMBL4740830 (MPO) | 64.54 ± 4.90 | 65.97 ± 8.07 |
| CHEMBL4747269 (MPO) | 80.81 ± 3.90 | 75.43 ± 6.62 |
| CHEMBL4751166 (MPO) | 72.51 ± 4.60 | 73.14 ± 0.24 |
| CHEMBL4453728 (MPO) | 56.21 ± 0.57 | 57.76 ± 0.35 |
| CHEMBL3670668 (HDAC6) | 81.74 ± 4.64 | 76.31 ± 1.67 |
| CHEMBL2018446 (HDAC6) | 95.49 ± 2.52 | 83.17 ± 0.57 |
| CHEMBL1879463 (HDAC6) | 74.27 ± 0.58 | 72.68 ± 0.36 |
| CHEMBL99 (HDAC6) | 80.30 ± 1.68 | 65.07 ± 0.55 |
| CHEMBL487253 (HDAC6) | 94.93 ± 0.32 | 60.59 ± 1.83 |
| CHEMBL511749 (HDAC6) | 76.55 ± 2.72 | 73.56 ± 1.51 |
| CHEMBL4098975 (HDAC6) | 89.38 ± 4.12 | 82.70 ± 0.71 |
| CHEMBL4790231 (MPO) | 72.32 ± 7.63 | 78.10 ± 2.74 |

p-value = 0.978501

p-value = 0.005412

b)

a)

**Figure S1**: Box plot chart with comparison of the molecular docking scores obtained for the specific reference compounds and candidate compounds for (a) MPO and (b) HDAC6.

b)

a)

p-value = 0.2141

p-value = 0.290896

**Figure S2**: Box plot chart with comparison of the molecular docking scores obtained for the global reference compounds and candidate compounds for (a) MPO and (b) HDAC6.

**Figure S3**: Best poses obtained with target HDAC6 for the molecules: A)- **3**; B)- **4**; C)- **6**; D) **8**; E)- **10**; F)- **11**; G)- **15** and H)- **16**. Hydrogen bonds are depicted in dark blue dashed lines.

**Table S11**: Ligand interactions with the amino acid residues of target HDAC6.

| Candidate | Fitness score | Residue | Interaction type | Distance (Å) |
| --- | --- | --- | --- | --- |
| 3 | 92.86 | Asp567 | Conventional Hydrogen Bond | 2.58 |
|  |  | His500 | Carbon Hydrogen Bond / Pi-Pi T-shaped | 2.75 / 4.55 |
|  |  | Phe680 | Pi-donor hydrogen bond / Pi- Stacked / Pi- Stacked | 3.14 / 5.25 / 3.74 |
|  |  | His499 | Carbon Hydrogen Bond / Pi -Alkyl | 2.50 / 4.28 |
|  |  | Phe620 | Pi-Pi stacked | 4.94 |
|  |  | Met682 | Pi -Alkyl | 5.15 |
|  |  | Pro501 | Pi-Alkyl / Alkyl / Pi-Alkyl / Pi-Alkyl | 3.91 / 4.70 / 4.99 / 5.33 |
|  |  | Pro748 | Pi -Alkyl | 4.79 |
|  |  | Leu749 | Alkyl / Alkyl / Pi-Alkyl | 3.81 / 4.59 / 4.87 |
| 4 | 91.47 | Ser568 | Conventional Hydrogen Bond | 2.00 |
|  |  | Phe679 | pi-sigma / Pi-Pi T-shaped / Pi-alkyl | 2.91 / 4.78 / 5.05 |
|  |  | Pro501 | pi-sigma / Pi -Alkyl | 2.34 / 5.21 |
|  |  | Phe680 | Pi-Pi stacked / Pi-Pi stacked / Pi-Pi T-shaped | 4.92 / 5.65 / 5.11 |
|  |  | His651 | Pi-Pi stacked | 4.99 |
|  |  | Met682 | Pi -Alkyl | 5.25 |
|  |  | Leu749 | Alkyl / Alkyl / Pi-Alkyl / Pi-Alkyl | 4.45 / 4.73 / 4.77 / 5.30 |
|  |  | His499 | Pi -Alkyl | 4.31 |
|  |  | Pro748 | Pi -Alkyl | 4.14 |
| 6 | 70.59 | Ser568 | Conventional Hydrogen Bond | 2.11 |
|  |  | His651 | Pi-cation / pi-cation | 3.88 / 2.97 |
|  |  | Phe620 | Pi-Pi stacked / Pi-alkyl | 4.18 / 5.40 |
|  |  | Phe680 | Pi - Alkyl | 4.19 |
|  |  | Leu749 | Pi - Alkyl | 4.37 |
|  |  | Met682 | Pi - Alkyl / Pi - Alkyl | 4.77 / 4.20 |
|  |  | Asp567 | Carbon Hydrogen Bond | 2.45 |
| 8 | 68.93 | Asp567 | Conventional Hydrogen Bond | 2.66 |
|  |  | Ser568 | Conventional Hydrogen Bond / Conventional Hydrogen Bond | 2.08 / 2.57 |
|  |  | phe680 | Pi-Pi stacked / Pi-Pi stacked | 3.98 / 5.27 |
|  |  | phe620 | Pi-Pi stacked / Pi - Alkyl | 4.86 / 4.93 |
|  |  | phe679 | Pi-Pi stacked / Pi - Alkyl | 4.41 / 4.83 |
|  |  | met682 | Pi - Alkyl / Pi - Alkyl | 5.36 / 4.31 |
|  |  | his5651 | Pi - Alkyl | 3.37 |
|  |  | leu749 | Alkyl / Pi - Alkyl | 4.84 / 5.11 |
|  |  | tyr782 | Pi - Alkyl | 4.83 |
| 10 | 76.88 | Zn2+ | Metal-Acceptor | 2.67 |
|  |  | Phe680 | Conventional Hydrogen Bond | 2.20 |
|  |  | His651 | Conventional Hydrogen Bond / Pi-Cation / Pi-Sulfur | 2.61 / 3.71 / 4.46 |
|  |  | Leu749 | Carbon Hydrogen Bond / Alkyl | 2.47 / 3.99 |
|  |  | Gly619 | Carbon Hydrogen Bond | 2.67 |
|  |  | Met682 | Pi-sulfur | 5.9 |
|  |  | Phe620 | Pi-Pi stacked / Pi-Pi stacked | 4.39 / 5.73 |
|  |  | His611 | Pi - Alkyl | 4.27 |
|  |  | His610 | Pi - Alkyl | 4.53 |
|  |  | Pro608 | Alkyl | 4.77 |
|  |  | Phe679 | Pi - Alkyl | 5.06 |
| 11 | 68.8 | Zn2+ | Metal-Acceptor | 2.05 |
|  |  | His651 | Conventional Hydrogen Bond / Pi-cation / Pi-Pi T-shaped | 2.98 / 2.98 / 4.96 |
|  |  | Tyr782 | Conventional Hydrogen Bond | 1.78 |
|  |  | Phe679 | Pi-Pi stacked / Pi-Pi T-shaped | 4.26 / 4.39 |
|  |  | Phe680 | Pi-Pi stacked | 3.79 |
|  |  | Phe620 | Pi-Pi stacked | 4.02 |
|  |  | Leu749 | Conventional Hydrogen Bond / Alkyl / Pi-alkyl | 2.59 / 5.44 / 4.83 |
| 15 | 75.46 | His651 | Conventional Hydrogen Bond | 1.88 |
|  |  | Met682 | Conventional Hydrogen Bond / Pi-alkyl / Pi-alkyl | 2.37 / 4.99 / 4.26 |
|  |  | Asp567 | Pi-Anion | 3.95 |
|  |  | Phe679 | Pi-Pi T-shaped | 4.63 |
|  |  | Phe620 | Pi-Pi stacked | 4.87 |
|  |  | Phe680 | Pi-donor hydrogen bond / Pi-Pi stacked / Pi-Pi stacked | 2.86 / 3.79 / 5.81 |
| 16 | 81.25 | Zn2+ | Metal-Acceptor | 2.30 |
|  |  | Tyr782 | Conventional Hydrogen Bond | 1.73 |
|  |  | His611 | Conventional Hydrogen Bond | 2.71 |
|  |  | Gly619 | Conventional Hydrogen Bond / Conventional Hydrogen Bond | 1.78 / 2.91 |
|  |  | His610 | Conventional Hydrogen Bond | 2.61 |
|  |  | Ser568 | Conventional Hydrogen Bond | 1.93 |
|  |  | His651 | Pi-cation | 3.85 |
|  |  | Asp567 | P-Anion | 3.87 |
|  |  | Phe680 | Pi-stacked / Pi-stacked | 4.69 / 4.61 |
|  |  | Phe620 | Pi-stacked | 4.26 |
|  |  | Leu749 | Alkyl | 4.20 |

**Figure S4**: Best poses obtained with target MPO for the molecules: A)- **3**; B)- **4**; C)- **6**; D) **8**; E)- **10**; F)- **11**; G)- **15** and H)- **16**. Hydrogen bonds are depicted in dark blue dashed lines.

**Table S12**: Ligand interactions with the amino acid residues of target MPO.

| Candidate | Fitness score | Residue | Interaction type | Distance (Å) |
| --- | --- | --- | --- | --- |
| 3 | 87.94 | Ser149 | Conventional Hydrogen Bond | 2.17 |
|  |  | Pro101 | Conventional Hydrogen Bond | 2.75 |
|  |  | Met411 | Pi-sigma / Pi-sulfur | 3.59 / 3.11 |
|  |  | Arg239 | Pi-cation / Pi-Alkyl | 4.05 / 4.99 |
|  |  | Hec601 | Pi-Anion / Pi-Pi Stacked / Pi-Alkyl | 3.38 / 3.87 / 4.42 |
|  |  | Glu102 | Carbon Hydrogen Bond | 2.07 |
|  |  | Arg424 | Pi-Cation | 4.79 |
|  |  | Phe147 | Pi-Pi Stacked / Pi-Alkyl | 4.53 / 4.45 |
|  |  | Phe99 | Pi-Pi T-Shaped / Pi-Alkyl | 4.44 / 5.25 |
|  |  | Glu116 | Carbon Hydrogen Bond | 2.07 |
| 4 | 86.49 | His95 | Conventional Hydrogen Bond | 3.06 |
|  |  | Arg239 | Pi-cation / Pi-Alkyl / Pi-Alkyl / Pi-Alkyl | 4.18 / 4.52 / 5.09 / 5.18 |
|  |  | Arg333 | Pi-Cation | 4.74 |
|  |  | Asp214 | Pi-Anion | 2.67 |
|  |  | Thr238 | Pi-sigma | 3.44 |
|  |  | Phe99 | Pi-Pi T-Shaped / Pi-Alkyl | 5.86 / 5.41 |
|  |  | Hec601 | Pi-Pi Stacked / Pi-Pi Stacked / / Pi-Alkyl / Pi-Alkyl / Pi-Alkyl | 3.43 / 4.93 / 4.05 / 4.27 / 5.20 |
|  |  | Leu216 | Pi-Alkyl | 5.21 |
|  |  | Cys232 | Alkyl / Pi-Alkyl | 4.16 / 5.34 |
|  |  | Pro220 | Alkyl | 5.00 |
|  |  | Phe366 | Pi-Alkyl | 5.29 |
|  |  | Glu102 | Pi-Anion | 3.31 |
| 6 | 68.70 | Arg239 | Conventional Hydrogen Bond / Pi-Alkyl / Pi-Alkyl | 2.88 / 4.80 / 4.73 |
|  |  | His95 | Conventional Hydrogen Bond | 2.62 |
|  |  | Gln91 | Conventional Hydrogen Bond | 2.92 |
|  |  | Arg424 | Pi-Cation | 4.21 |
|  |  | Hec601 | Carbon Hydrogen Bond / Pi-Pi Stacked^5^ / Alkyl / Pi-Alkyl / Pi-Alkyl | 2.48 / 3.39 / 3.19 / 4.47 / 5.18 / 4.92 / 4.08 / 4.40 / 4.54 |
| 8 | 73.30 | Hec601 | Conventional Hydrogen Bond / Pi-Pi Stacked^3^ / Pi-Alkyl / Pi-Alkyl | 1.9 / 4.42 / 3.30 / 5.10 / 4.81 / 4.38 |
|  |  | Arg239 | Conventional Hydrogen Bond / Pi-Alkyl / Pi-Alkyl | 2.74 / 5.40 / 4.68 |
|  |  | Phe366 | Pi-Alkyl | 4.56 |
|  |  | Pro220 | Alkyl | 4.64 |
|  |  | Leu415 | Pi-Alkyl | 5.13 |
| 10 | 82.28 | Thr238 | Pi-lone pair | 2.54 |
|  |  | Arg239 | Pi-Cation / Alkyl / Pi-Alkyl^3^ | 3.84 / 4.88 / 5.08 / 4.62 / 5.11 |
|  |  | Hec601 | Pi-Cation / Pi-Anion / Pi-Pi Stacked^4^ / Alkyl / Pi-Alkyl^2^ | 4.28 / 3.30 / 3.36 / 3.17 / 4.74 / 4.99 / 4.68 / 4.30 / 4.44 |
|  |  | Cys232 | Pi-Alkyl | 5.18 |
|  |  | Glu102 | Carbon Hydrogen Bond | 2.85 |
|  |  | Se4r149 | Carbon Hydrogen Bond | 2.12 |
| 11 | 70.92 | His95 | Conventional Hydrogen Bond | 2.38 |
|  |  | Arg239 | Conventional Hydrogen Bond / Pi-Alkyl | 3.32 / 4.42 |
|  |  | Glu102 | Carbon Hydrogen Bond / Pi-Anion | 2.30 / 2.58 |
|  |  | Hec601 | Pi-sigma / Pi-Pi Stacked | 3.99 / 4.03 |
|  |  | Leu415 | Pi-sigma | 3.92 |
|  |  | Phe99 | Pi-sulfur | 5.07 |
|  |  | Leu420 | Pi-alkyl | 5.17 |
|  |  | Pro145 | Pi-alkyl | 5.42 |
| 15 | 83.93 | Hec601 | Conventional Hydrogen Bond / Pi-sigma / Pi-Pi Stacked^3^ / Pi-alkyl^5^ | 2.61 / 1.99 / 2.97 / 3.75 / 5.00 / 4.76 / 5.13 / 4.11 / 4.17 / 4.18 |
|  |  | Gln91 | Conventional Hydrogen Bond | 3.31 |
|  |  | His95 | Pi-alkyl | 3.66 |
|  |  | Arg239 | Pi-alkyl^2^ | 4.73 / 4.94 |
|  |  | Pro145 | Pi-alkyl | 5.45 |
|  |  | Leu415 | Pi-alkyl | 5.33 |
|  |  | Leu420 | Pi-alkyl | 5.24 |
|  |  | Glu102 | Carbon Hydrogen Bond / Pi-anion | 2.41 / 2.53 |
|  |  | Asp94 | Carbon Hydrogen Bond | 2.72 |
| 16 | 79.74 | Arg239 | Conventional Hydrogen Bond / Carbon hydrogen bond / Pi-cation / Amide-Pi Stacked / Pi-alkyl^2^ | 3.28 / 3.33 / 4.86 / 5.29 / 5.35 / 3.96 |
|  |  | His95 | Conventional Hydrogen Bond | 2.91 |
|  |  | Thr238 | Pi-lone pair | 2.56 |
|  |  | Phe366 | Pi-Pi T-Shaped | 5.02 |
|  |  | Met411 | Alkyl | 5.12 |
|  |  | Hec601 | Pi-alkyl | 4.09 |
|  |  | Cys232 | Alkyl / Pi-Alkyl | 3.95 / 4.14 |
|  |  | Leu216 | Alkyl / Pi-Alkyl | 4.44 / 5.30 |

**Table S13**: Structural identification (IUPAC, SMILES) and physicochemical characterization of potential dual-target ligands obtained from the PubChem database.

| Physicochemical properties | Molecule 11 | Molecule 16 |
| --- | --- | --- |
| Molecular Weight | 368.4 g/mol | 417.5 g/mol |
| Hydrogen Bond Donor Count | 2 | 3 |
| Hydrogen Bond Acceptor Count | 5 | 4 |
| Rotatable Bond Count | 3 | 5 |
| XLogP3-AA | 1.5 | 3 |
| Topological Polar Surface Area | 116 Å² | 98.7 Å² |
| SMILES | O=C(NO)c1ccc(C(=O)N2CCn3c(-c4ccccc4)cnc3C2)s1 | Cc1ccc(C(=O)Nc2cc(C(=O)N(C)C)cc(-c3ccc(C(=O)NO)cc3)c2)cc1 |
| IUPAC name | N-hydroxy-5-(3-phenyl-6,8-dihydro-5H-imidazo[1,2-a]pyrazine-7-carbonyl)thiophene-2-carboxamide | 3-[4-(hydroxycarbamoyl)phenyl]-N,N-dimethyl-5-[(4-methylbenzoyl)amino]benzamide |
